# Using historical genome‐wide DNA to unravel the confused taxonomy in a songbird lineage that is extinct in the wild

**DOI:** 10.1111/eva.13149

**Published:** 2020-11-07

**Authors:** Pratibha Baveja, Kritika M. Garg, Balaji Chattopadhyay, Keren R. Sadanandan, Dewi M. Prawiradilaga, Pramana Yuda, Jessica G. H. Lee, Frank E. Rheindt

**Affiliations:** ^1^ Department of Biological Sciences National University of Singapore Singapore Singapore; ^2^ Institute of Bioinformatics and Applied Biotechnology Bangalore India; ^3^ Max Planck Institute for Ornithology Seewiesen Germany; ^4^ Bidang Zoologi Puslit Biologi – LIPI Cibinong Indonesia; ^5^ Fakultas Teknobiologi Universitas Atma Jaya Yogyakarta Yogyakarta Indonesia; ^6^ Department of Conservation and Research Wildlife Reserves Singapore Singapore Singapore

**Keywords:** Asian Pied Starling, Asian Songbird Crisis, conservation genetics, museum samples, South‐East Asia, target enrichment, wildlife trade

## Abstract

Urgent conservation action for terminally endangered species is sometimes hampered by taxonomic uncertainty, especially in illegally traded animals that are often cross‐bred in captivity. To overcome these problems, we used a genomic approach to analyze historical DNA from museum samples across the Asian Pied Starling (*Gracupica contra*) complex in tropical Asia, a popular victim of the ongoing songbird crisis whose distinct Javan population (“Javan Pied Starling”) is extinct in the wild and subject to admixture in captivity. Comparing genomic profiles across the entire distribution, we detected three deeply diverged lineages at the species level characterized by a lack of genomic intermediacy near areas of contact. Our study demonstrates that the use of historical DNA can be instrumental in delimiting species in situations of taxonomic uncertainty, especially when modern admixture may obfuscate species boundaries. Results of our research will enable conservationists to commence a dedicated ex situ breeding program for the Javan Pied Starling, and serve as a blueprint for similar conservation problems involving terminally endangered species subject to allelic infiltration from close congeners.

## 1 INTRODUCTION

Conservation action is ideally based on adequate scientific data (Rose et al., 2019). A lack of clarity over species boundaries may often lead to failed conservation action (Ely et al., 2017). Critically endangered taxa, or those extinct in the wild, pose a particular dilemma, as surviving individuals are often inaccessible to taxonomists and susceptible to genetic admixture, making historical museum material the only reliable source of taxonomic information (Payne & Sorenson, 2002). With the emergence and continuous advancement of next‐generation sequencing and related bioanalytical pipelines (Derkarabetian et al., 2019; Linck et al., 2017; Prüfer, 2018; Rowe et al., 2011; Schubert et al., 2014; Schuster, 2007), it is now possible to study trace amounts of DNA from historical samples, thereby overcoming the problems of low sample size (Nedoluzhko et al., 2020; Tsai et al., 2019), modern genetic admixture (Prost et al., 2020) and unavailability of live individuals (Wood et al., 2018).

**FIGURE 1:**
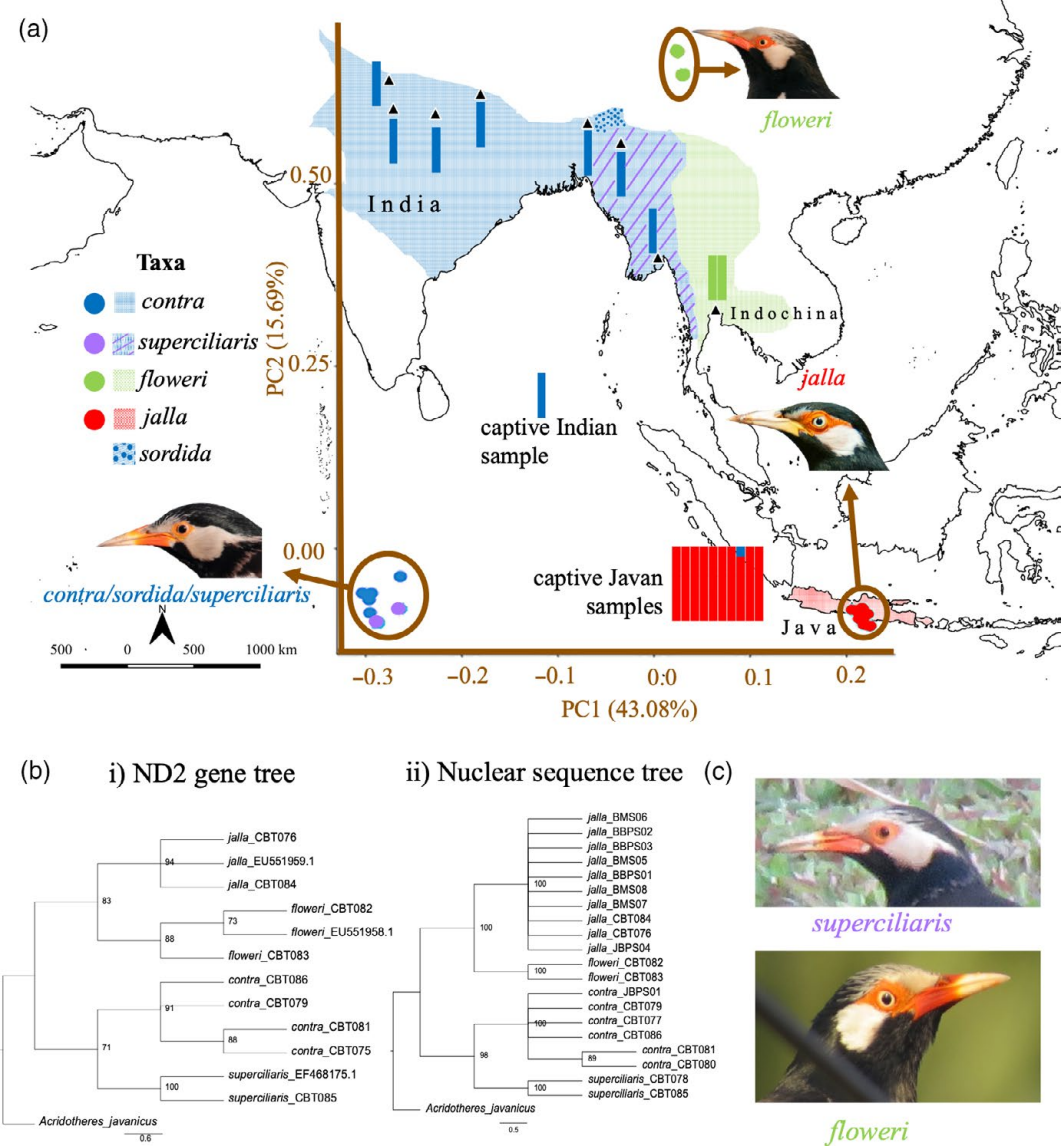
(a) Map showing the Asian distribution, sampling localities, and population structure for the five taxa of the Asian Pied Starling (*Gracupica contra*) complex: nominate *contra*, *sordida*, *superciliaris*, *floweri* and *jalla*. Black triangles depict wild sampling localities. Principal component analysis (PCA) is superimposed on the map with brown axes and based on an unlinked variant set of 936 SNPs (SNP set A2). STRUCTURE results are displayed as colored columns adjacent to collection localities (one column per individual) and are based on 412 unlinked SNPs (SNP set B2) at *K* = 3, with each color representing a different ancestral contribution. Legend depicts the color of PCA points (circles) and of distribution ranges (squares) for each taxon. Coastline map sourced from Natural Earth (www.naturalearthdata.com). Bird photo credits: *contra*—Mustafa Sozen, *floweri*—Peter Ericsson, *jalla*—Boas Emmanuel. (b) Maximum‐likelihood consensus trees based on (i) mitochondrial ND2 gene with bootstrap support ≥70% shown beside nodes and (ii) concatenated nuclear sequences of 117 loci (each with >12 SNPs per locus) with bootstrap support ≥95% shown beside nodes. GenBank accession numbers or sample codes are given behind each taxon name. (c) Pictures of *superciliaris* and *floweri* demonstrating differences in eye color and extent of bare facial skin across the two taxa. Photo credits: *superciliaris*—Thomas Brooks, *floweri*—Tom Wheatley

The Asian Pied Starling (*Gracupica contra*) in South and South‐East Asia (Figure 1a, Craig et al., 2020) is one such species complex in which both taxonomic uncertainty and the threat of genomic infiltration have hampered conservation. Traditionally, this complex has comprised five taxa, *contra*, *sordida*, *superciliaris*, *floweri,* and *jalla*, recognized at the subspecies level based on morphological differences (Figure 1a,c, Gill et al., 2020). The nominate subspecies *contra* from the Indian subcontinent and the widely synonymized subspecies *sordida* from Assam have a largely black crown and a reddish‐orange bill base, with extremely limited bare skin around the eye (Figure 1a). In contrast, neighboring *superciliaris* from Myanmar has white streaks on the forehead and a wider extent of orange bare skin around the eye (Figure 1c). The subspecies *floweri*, centered in Thailand, has even more extensive white streaking on the forehead and more bare facial skin (Figure 1a,c). Finally, the morphologically most distinctive subspecies *jalla*, originally from Java and Bali, lacks any reddish coloration on its bill base and sports the most extensive patch of deep‐orange bare skin around the eye (Figure 1a, Horsfield, 1821). Due to this distinctive morphology, it has sometimes been considered a separate species, the Javan Pied Starling (Birdlife International, 2020; del Hoyo et al., 2020).

The distinct subspecies *jalla* is extinct in the wild following unsustainable and illegal wildlife trade, and the few surviving captive populations are subject to an ever‐increasing market demand (Chng et al., 2015; Eaton et al., 2015; Marshall et al., 2019). Because of a lack of fresh recruitment of wild captures, and possibly in order to increase market value, illegally captive *jalla* individuals are frequently cross‐bred with other taxa, especially *floweri* from Thailand, compromising the genetic integrity of Javan Pied Starlings. At the recent Songbird Crisis Summit held by the IUCN Specialist Group for Asian Songbird Trade in Singapore in 2019, *jalla* was identified as one of the top 10 taxa warranting immediate conservation action to preclude final extinction. However, a full resolution of the taxonomic status of *jalla* and other taxa within the Asian Pied Starling complex has never been achieved, and the genetic integrity of some of the last remaining captive samples of *jalla* has so far been left unaddressed. As *jalla* is deemed critically endangered (Birdlife International, 2017) and pure *jalla* individuals must now be scarce in the markets, historical museum samples are the only credible source of DNA to resolve the identity and affinities of the various components of the Asian Pied Starling complex and design appropriate conservation strategies.

In this study, we used the Asian Pied Starling complex as a model to address the complicated conservation status of endangered organisms that are subject to taxonomic uncertainty and susceptible to an erosion of genomic integrity through allelic infiltration from closely related taxa. Our use of historical samples ensures that we can reach appropriate taxonomic conclusions regardless of modern admixture practices in captivity. We used a target‐enrichment approach to capture a genome‐wide array of loci for analysis (Chattopadhyay et al., 2019; McCormack et al., 2016; Wood et al., 2018). Our work can serve as a blueprint for future studies in which historical samples can shed light on similar taxonomically ambiguous cases to facilitate conservation action.

## 2 METHODS

### 2.1 Sample collection

We obtained 12 historical tissue samples (collection dates range from 1878 to 1983) from four subspecies of the Asian Pied Starling complex (*contra*, *jalla*, *superciliaris*, and *floweri*) from the American Museum of Natural History (AMNH), University of Michigan Museum of Zoology (UMMZ), and Royal Ontario Museum (ROM) (Table S1). We were unable to procure material for *sordida*, a subspecies that is thought to be geographically restricted to eastern Assam in northeast India, although we did include DNA of a specimen collected immediately adjacent to the traditionally accepted range of *sordida* in central Assam. However, note that *sordida'*s purported distinguishing features are doubtfully distinct from nominate *contra* (Ripley, 1950), and it is variably synonymized with *contra*. We also procured nine fresh blood samples (Table S1) across two subspecies (*contra* and *jalla*) through Bali Bird Park (Indonesia) and Jurong Bird Park (Singapore) whose identity was not in doubt as they displayed the typical morphological traits of these two distinct subspecies (Figure 1a). However, as the surviving captive population of *jalla* is known to be subject to breeding practices exposing birds to admixture, genomic comparison with historic DNA samples allows us to test their purity. We strictly adhered to the Institutional Animal Care and Use Committee's Biodiversity protocol B17‐0459 while carrying out our project.

### 2.2 DNA extraction

We used Qiagen DNeasy Blood and Tissue kits (Qiagen, Germany) for DNA extraction. The manufacturer's protocol was modified for the historical samples according to Chattopadhyay et al. (2019), and DNA extraction was carried out in a dedicated historical DNA facility, strictly isolated from post‐PCR workspace (Cooper & Poinar, 2000). All historical DNA extractions were carried out under sterile conditions in a separate Class II Biosafety cabinet in batches. Each batch had its own negative control to monitor contamination (Krings et al., 1997). We used the Qubit® 2.0 High Sensitivity DNA Assay (Invitrogen, USA) for DNA quantification. For historical samples, we additionally used an Agilent High Sensitivity DNA kit (Agilent Technologies, USA) for fragment analysis and detection of degraded DNA.

### 2.3 Library preparation

Library preparation of historical samples was carried out in a sterile environment in a biosafety cabinet with laminar airflow. The cabinet was located in an isolated space between the historical DNA clean facility and post‐PCR area. Historical samples were first subjected to NEB Next FFPE DNA Repair Mix (New England BioLabs) to repair degraded and damaged regions (Pääbo et al., 2004). DNA of fresh samples was first sheared using a Bioruptor Pico (Diagenode, Belgium) with 12 cycles of sonication (conditions: 30s ON and 30s OFF) to obtain an average fragment size of ~250 bp. Subsequently, we prepared dual indexed libraries using a NEB Next Ultra DNA Library Prep kit (New England BioLabs) for sheared fresh and FFPE‐treated historical DNA samples. NEB Next Multiplex Oligos for Illumina (New England BioLabs) were used for indexing each sample. As an additional safeguard against cross‐contamination, we carried out historical DNA library preparation in two batches; geographically proximate specimens were preferably processed in different batches to facilitate easy detection of cross‐contamination (Tonnis et al., 2005). We had separate negative controls for library preparation but we also carried forward negative controls from DNA extraction through library preparation to monitor contamination at every step of the laboratory work.

We performed DNA quantification and fragment analysis for all the sample DNA libraries and negative controls. DNA quantification and fragment analysis of negative controls revealed no quantifiable DNA but only primer–dimers and adapter sequences in the fragment profile.

### 2.4 Target locus design

Target regions for bait design were chosen to account for both recent and deep divergence within the Asian Pied Starling complex and hence comprised both variable intronic regions and conserved exons (Chattopadhyay et al., 2019). We used EvolMarkers (Li et al., 2012) to identify single‐copy exons (hence, filtering out paralogs) conserved across the genomes of the Javan Myna (*Acridotheres javanicus*, GCA_002849675.1), Collared Flycatcher (*Ficedula albicollis*, GCA_000247815.1), and Zebra Finch (*Taeniopygia guttata*, GCF_003957565.1). Javan Myna (Low et al., 2018) was chosen for its phylogenetic proximity to Asian Pied Starlings (Lovette et al., 2008; Lovette & Rubenstein, 2007; Zuccon et al., 2008). We set a minimum of 55% identity and an e‐value <10E‐15 for the BLAST (Altschul et al. 1990) search within the EvolMarkers database to identify conserved exons. Only exons >500 bp were used for downstream analysis. We used BEDtools 2.28.0 (Quinlan & Hall, 2010) to extend these targeted coding regions to include flanking intronic regions 500 bp upstream and downstream on the Javan Myna genome in order to account for shallow divergence. We further accounted for overlapping targets by merging overlapping loci (Quinlan & Hall, 2010). In addition, we only retained loci with 40%–60% GC content. Finally, we filtered for repeat regions in RepeatMasker 4.0.7 (Smit et al., 2015) to retain a total of 983 loci ranging from 1,502–8,173 bp in length. MYcoarray (USA) used these target loci to design 75,292 100‐bp‐long RNA baits with a 4X tiling density for in‐solution hybridization.

### 2.5 Sequence capture

We used these specifically designed RNA baits to enrich our previously generated DNA libraries with target‐specific endogenous loci through in‐solution hybridization capture. The myBaits protocol (myBaits manual version 3) was followed with modifications (Chattopadhyay et al., 2019). We used a Qubit® 2.0 High Sensitivity DNA Assay (Invitrogen, USA) to quantify the enriched libraries followed by fragment analysis (Agilent Technologies, USA). Samples were pooled at equimolar ratios and sent for paired‐end sequencing (Illumina HiSeq 4000 2 × 151 bp). Fresh and historical samples were sequenced separately.

### 2.6 Quality filtering

We checked the quality of the raw DNA sequence data using FastQC v0.11.7 (Babraham Bioinformatics). We used Trimmomatic v0.33 (Bolger et al., 2014) to remove adapters, reads with low quality (Phred score <20), and reads below a minimum length of 36 bp. Duplicate reads and low complexity reads were removed using FastUniq v1.1 (Xu et al., 2012) and fastp v0.20.0 (Chen et al., 2018), respectively. Subsequently, the quality of the reads was again assessed in FastQC v0.11.7 (Babraham Bioinformatics) to ensure a complete removal of adapters and a high sequence quality.

### 2.7 SNP‐calling pipeline for population‐genomic analysis

We mapped the cleaned reads to the target loci using BWA‐MEM v 0.7.12 (Li, 2013). The aligned sam files were converted to bam format using Samtools v1.9 (Li et al., 2009). Then, we added read groups to the bam files using AddOrReplaceReadGroups in PICARD v2.20.0 (Picard tools, Broad Institute, Cambridge, MA, USA) and marked duplicate reads using MarkDuplicates in PICARD v2.20.0. The reads were then realigned around indels using RealignerTargetCreator and IndelRealigner in GATK v3.8 (McKenna et al., 2010) to obtain improved local alignment. For historical samples, we processed the resultant files in mapDamage v.2.0.9 (Jónsson et al., 2013) to check for historical DNA‐specific damage patterns. When we compared the results for historical and fresh samples, we found no significant damage in the historical samples. We rescaled the quality scores for further downstream analyses. We sorted the final mapped reads for historical and fresh samples in Samtools v1.9 (Li et al., 2009) and assessed their quality in Qualimap v2.2.1 (García‐Alcalde et al., 2012). Finally, we obtained genotype likelihoods for the alignments using “mpileup” and called the variants from genotype likelihoods using the "call" utility in bcftools v1.9 (Li, 2011), obtaining a total of 43,050 SNPs. The variants were filtered iteratively using "vcffilter" in vcflib v1.0.0 (Garrison, 2012) and vcftools v0.1.16 (Danecek et al., 2011) to retain only reliable, well‐represented, paralog‐filtered, and high‐quality variants by means of the following parameters: minimum depth of 20 reads per individual, minor allele count 3, minimum quality 30, minor allele frequency 0.05, minimum mean depth 30, and maximum mean depth 175. We removed individuals with >50% missing data, losing three historical samples (2 *contra* from Assam and one *jalla*; Figure S1).

To check for contamination, we carried the DNA reads from negative controls through the above pipeline. After the above steps, final negatives (i.e., those that had been carried through to in‐solution hybridization) produced reads of three loci. In contrast, nonenriched negatives (i.e., those that had only been carried through library preparation) did not produce any reads mapping to target loci. This suggests that reads obtained from the DNA of final negatives were derived from nondegraded RNA baits added to negative controls during in‐solution hybridization. As a precautionary step, we removed these three loci from all analyses.

We generated multiple final variant sets to account for different biases. Firstly, variants were filtered to retain only those loci with <5% missing data, resulting in a total of 6333 SNPs (SNP set A1) across 18 individuals. Secondly, to account for linkage disequilibrium between SNPs located on the same locus, we selected one random SNP from each locus and retained a final SNP set of 936 variants (SNP set A2). To further check for linkage across loci, we ran PLINK v1.9 (Chang et al., 2015) with the following parameters: sliding window of 50 SNPs, step size of 5, and pairwise linkage disequilibrium <0.1. We did not detect evidence for linkage disequilibrium between our filtered variants even at such strict parameters.

Our quality filters for missing data led to a loss of both historical samples from central Assam (northeast India), collected from close to the range of *sordida* (from eastern Assam) and possibly containing an allelic profile representative of the unsampled subspecies *sordida*. Therefore, to account for this bias, we constructed another SNP set allowing for no missingness in loci and keeping all other parameters equal. This alternative SNP‐calling regime resulted in a set of 871 SNPs (SNP set B1) across 20 individuals. To account for linkage, we again chose one random SNP from each locus, resulting in a total of 412 SNPs (SNP set B2) while managing to salvage one historical sample from Assam and one historical sample of *jalla*. Last, we constructed a third SNP set only containing a selection of loci that displayed the highest levels of polymorphism; that is, each locus contained >20 SNPs to examine whether these highly divergent loci carry an underlying genomic signal that corroborates the genome‐wide phylogenetic signal. For this final set, we obtained a total of 749 SNPs (SNP set C) across 25 loci.

We investigated potential pairwise kinship relationships among individuals within the same population using SNPRelate (Zheng et al., 2012) and checked for outlier loci under positive selection across all SNP sets using Bayescan v2.1 (Fischer et al., 2011) at a false discovery rate of 5%. We did not detect kinship or positive selection in any of our variant sets.

### 2.8 Population‐genomic analyses

We used the R package SNPRelate (Zheng et al., 2012) in RStudio v1.0.143 (RStudio Team, 2015) to construct principal component analysis (PCA) plots to visualize population structure based on all sets of linked and unlinked SNPs (A1, A2, B1, B2, and C). We further investigated population structure using the Bayesian population clustering program STRUCTURE (Pritchard et al., 2000) and associated programs Structure Harvester (Earl, 2012) and CLUMPP (Jakobsson & Rosenberg, 2007). We ran 10 replications of our unlinked SNP sets A2 and B2 for a population cluster size of *K* = 1–6 using a burn‐in period of 200,000 and 1,000,000 Monte Carlo Markov chain iterations for each replicate.

**FIGURE 2:**
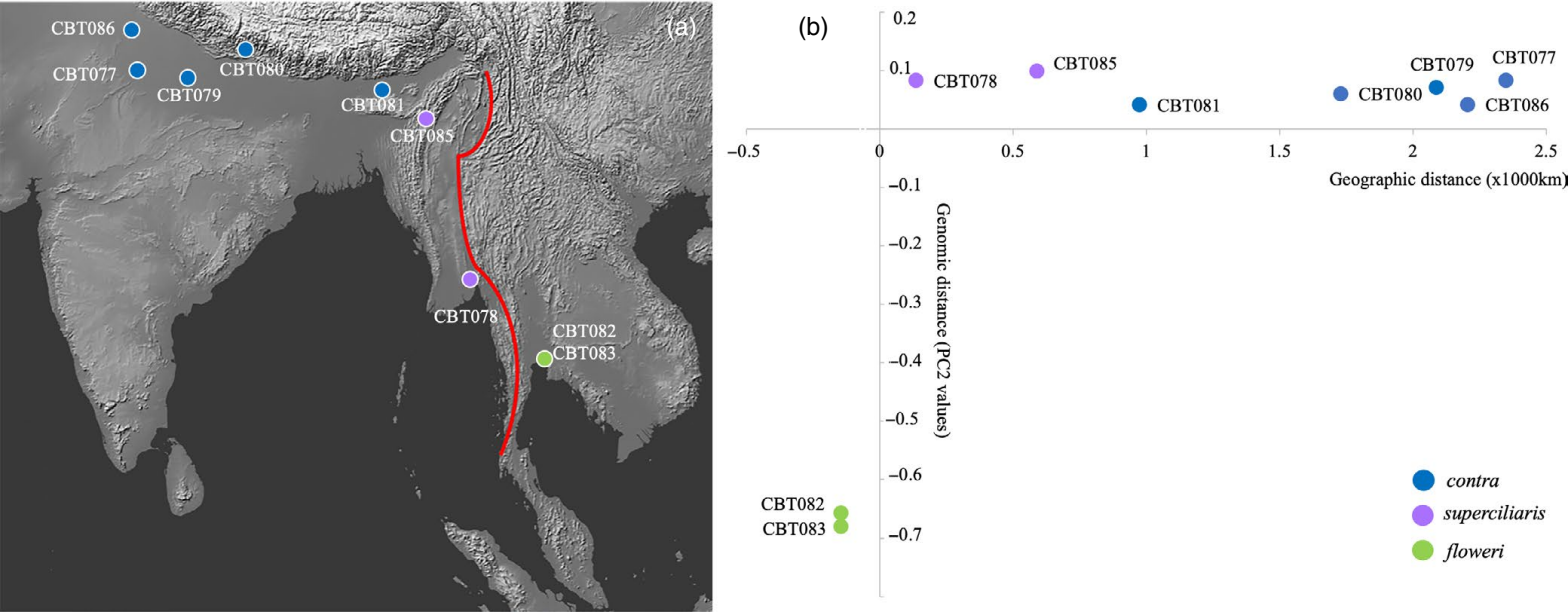
(a) Terrain map of South and South‐East Asia. Each individual point marks a wild sample locality and the red line depicts the terrain boundary of the Thai–Burmese border mountain range, which separates taxa *superciliaris* (from Myanmar) and *floweri* (from Thailand). Terrain map sourced from Natural Earth (www.naturalearthdata.com); (b) Graph showing relationship between geographic and genomic distances. The *x*‐axis refers to the geographic distance of the collection localities of *contra*, *superciliaris,* and *floweri* to the nearest point along the terrain boundary (red line in panel a), with positive values reflecting points west of the terrain boundary and negative values east of the boundary. The *y*‐axis reflects genomic distance expressed in terms of PC2 values from the PCA plot based on SNP set B2 (Fig. S3b)

We performed an analysis to inspect the relationship between geographic distance and genomic distance specifically across the boundary between the two adjacent taxa *floweri* and *superciliaris*. We first visualized their boundary along the Thai–Burmese border mountain range (Tenasserim Hills, Dawna range, and Karen Hills) on a terrain map obtained from Natural Earth (www.naturalearthdata.com; Figure 2a). We then measured the geographic distances of the collection localities of all mainland Asian individuals (including *contra*, *superciliaris*, and *floweri*) from the line marking the terrain boundary and plotted these geographic distances against the PC2 values of our PCA plot from SNP set B2, which is representative of mainland Asian genomic differentiation in Asian Pied Starlings. The insular taxon *jalla* was excluded from this analysis as its distribution is widely disjunct and not geographically contiguous.

### 2.9 Morphological inspection

We performed a morphological inspection of Asian Pied Starling photographic material from Thailand and Myanmar deposited on two online photo repositories, Oriental Bird Images (www.orientalbirdimages.org) and eBird. (www.ebird.org). We only included adult birds and we eliminated replicate pictures of identical birds posted multiple times. We counted multiple birds in the same picture separately. For the taxon *floweri* from Thailand, we examined a total of 13 birds on Oriental Bird Images and a total of 318 birds on eBird. For the taxon *superciliaris* from Myanmar, we examined one bird on Oriental Bird Images and a total of 23 birds on eBird. We focused on two important morphological traits during inspection: (a) extent of bare red/orange facial skin and (b) eye color. If the length of bare facial skin extending from the posterior end of the eye toward the nape was greater than the length of bare skin extending from the anterior end of the eye to the base of the bill, the bare facial skin patch was considered large, and vice versa. The eye color was examined to check whether it was pale or dark. Traits were recorded as “indeterminate” in photographs of insufficient quality or those in which the bird was too distant or depicted at an unsuitable angle.

### 2.10 Sequence assembly for phylogenetic analysis

We assembled the cleaned DNA reads using HybPiper v1.3.1 (Johnson et al., 2016) to obtain sequences of genomic target loci across all individuals. We also assembled reads for the mitochondrial gene ND2 obtained as a nonspecific product from target‐enrichment sequencing, adding sequences available from the NCBI database (https://www.ncbi.nlm.nih.gov/; EF468175.1) as a reference.

We ran the python script *reads_first.py* from HybPiper v1.3.1, specifying “bwa” for mapping (Li, 2013) and inputting a “cov_cutoff” value of 18 for subsequent de novo assembly at each locus (Bankevich et al., 2012). We ignored HybPiper's translation output for exons. We also ran the above pipeline for the DNA output from our negative controls, which produced sequence for three loci, probably due to nondegraded RNA baits (see above). Again, we excluded these three loci from all further analyses. We used *get_seq_lengths.py* to summarize the total loci recovered and visualized this information using *gene_recovery_heatmap.R* in RStudio v1.0.143 (RStudio Team, 2015). We also ran *hybpiper_stats.py* to summarize target enrichment and locus recovery efficiency across samples (Table S2). As there was limited locus recovery (~23%) for one historical *contra* sample (CBT075) due to poor sequence quality (Table S2), we excluded this sample from further analyses. We also excluded 15 nuclear loci that were not retrieved across all samples. In summary, we retrieved a total of 965 nuclear loci for 20 samples and recovered the mitochondrial ND2 gene for 12 samples. We then used the python script *retrieve_sequences.py* in HybPiper v1.3.1 (Johnson et al., 2016) to collate sequences from all individual fasta files as a multifasta file for each locus.

### 2.11 Tree construction

We constructed gene trees for all nuclear sequence loci containing >20 SNPs in RAxML‐NG v.0.9.0 (Kozlov et al., 2019). This resulted in 24 individual gene trees (minus one gene that was excluded due to missing data during HybPiper assembly). We also constructed a gene tree for the mitochondrial gene ND2 using all samples in which the recovery length was >960 bp. We supplemented our dataset with mitochondrial sequences available on the NCBI database (EF468175.1, EU551958.1, EU551959.1). We used sequences from the NCBI database belonging to the Javan Myna (*Acridotheres javanicus*) as an outgroup (nuclear loci—GCA_002849675.1, ND2—EU403593.1).

The resultant multifasta files were aligned in MAFFT v7.388 (Katoh & Standley, 2013). We then used Phyutility v.2.2.6 (Smith & Dunn, 2008) to clean aligned multifasta files removing sites with >50% missing data. We ran jModelTest v.2.1.10 (Darriba et al., 2012) to identify the best nucleotide substitution model for each locus based on the corrected Akaike information criterion (AICc). Finally, we constructed maximum‐likelihood (ML) gene trees in RAxML‐NG v.0.9.0 (Kozlov et al., 2019) using the best nucleotide substitution model as identified by jModelTest (Table S3) with 5,000 bootstrap iterations. Trees were visualized in FigTree v1.4.4 (Rambaut, 2018). We additionally computed uncorrected pairwise distances for the ND2 gene in MEGA7 (Kumar et al., 2016).

To concentrate phylogenetic signal, we selected all our sequence loci with >12 SNPs per locus to construct a concatenated sequence tree across constituent taxa, resulting in an alignment of 299,371 bp consisting of a total of 117 loci for which sequences had been assembled through HybPiper. We obtained the outgroup sequence for these loci from the Javan Myna genome on NCBI (GCA_002849675.1). We first aligned the multifasta files for each locus (Katoh & Standley, 2013) and cleaned the aligned multifasta files to remove sites with >50% missing data (Smith & Dunn, 2008). We concatenated the resultant files in SequenceMatrix (Vaidya et al., 2011) and exported the concatenated file in phylip format. We constructed an ML tree for the concatenated sequence file in RAxML‐NG v.0.9.0 (Kozlov et al., 2019) using the general time‐reversible (GTR) model with 5,000 bootstrap iterations. The tree was visualized in FigTree v1.4.4 (Rambaut, 2018).

### 2.12 Gene ontology

For the 25 selected nuclear sequence loci (i.e., those with >20 SNPs per locus), we further performed gene ontology (GO) enrichment tests to identify whether functional loci associated with molecular, biological, or cellular processes might be particularly represented in these stretches of sequence. We annotated the entire set of target loci using Blast2GO (Conesa et al., 2005). We then performed the GO enrichment test in Blast2GO for the 25 selected loci using—as a reference set—all 980 loci excluding the three loci that had been removed from analyses because of negative control bias (see above). The test was performed using corrected *p*‐values at a false discovery rate of .05. We also performed over‐representation analysis (ORA) using the web‐based tool WebGestalt (Zhang et al., 2005).

## 3 RESULTS

We found no significant DNA damage in our historical samples, with a deamination incidence at 0%–1% at read termini (Table S4). Based on the number of reads that mapped to their target compared to the total number of reads, target‐enrichment efficiency ranged between 24%–60% for historical samples (except for two poor‐quality samples) and 58%–75% for fresh samples (Table S2). Based on the number of loci retrieved compared to the total number of target loci, target‐enrichment efficiency was >98% except for one poor‐quality historical sample of *contra* that was excluded from further analysis (Table S2). The length of loci recovered was >80% of total locus length for most samples except for a few degraded historical samples (Figure S2).

PCAs generally indicated three genomic clusters within the Asian Pied Starling complex; *contra* and *superciliaris* were genomically proximate within one cluster, whereas *floweri* and *jalla* formed two distinct clusters (Figure 1a, Figure S3a–c). For both *jalla* and *contra*, the contemporary and historical samples of each taxon formed a single spatial unit, confirming that our dataset did not suffer from ancient DNA artifacts and indicating an absence of admixture in our contemporary samples (Figure 1a, Figure S3a–c).

STRUCTURE analysis revealed the same tripartition of Asian Pied Starlings across all variant sets even at *K* = 4, 5, and 6 (Figure 1a, Figure S4). When enforcing *K* = 2, *contra* and *superciliaris* formed one genomic division and *jalla* formed another genomic division, whereas *floweri* displayed genomic admixture between these two groups with a slightly greater proportion of a *jalla* contribution in its genome (Figure S4). One historical *jalla* sample, CBT076, showed traces of potential *contra* admixture in its STRUCTURE profile at *K* = 3 (Figure 1a). However, we interpret this pattern as a likely artifact due to heavy DNA damage as this was one of the samples with the poorest DNA quality (high missing data; Figure S1), which was specifically salvaged in SNP set B. The three distinct genomic Pied Starling clusters became less well defined, with more blurred boundaries, when only selected loci with a high SNP count (>20 SNPs per locus) were analyzed (Figure S3d).

We constructed gene trees for the 25 loci with the highest SNP count (>20 variants per locus) to investigate whether loci of high divergence carry a phylogenetic signal that reflects the overarching genome‐wide phylogenetic signal. In general, the phylogenetic signal across these 25 loci was mutually incongruent, and we failed to identify cohorts of candidate loci that would have converged on a particular phylogenetic signal—whether equal or different from the full dataset's signal (Figure S5). Some gene trees retrieved high support for a distinct clade of *jalla*, explaining its separation in the PCA of SNP set C (Figure S3d). To examine whether this distinct placement of *jalla* may be driven by adaptive processes reflected in a subset of loci, we carried out GO enrichment tests and ORA analyses for these selected loci. We found no statistically significant GO terms (Figure S6; Tables S5 and S6), which is consistent with expectations from our low locus sample size (Mishra et al., 2014). Some of the biological processes that were most frequently identified as overrepresented among the genes adjacent to our candidate loci include metabolic processes and biological regulation (Figure S7).

All four taxa analyzed emerged as monophyletic lineages with strong support in both the concatenated nuclear tree and the mitochondrial ND2 tree (Figure 1b). Geographic distances notwithstanding, the taxon *floweri* emerged as sister to *jalla*, not to *contra/superciliaris* (Figure 1b). In accordance with the population‐genetic results, the taxon *superciliaris* emerged as most closely related to *contra* in the concatenated nuclear tree (Figure 1b). The mitochondrial ND2 sequence divergence between the *superciliaris‐contra* cluster and the *floweri‐jalla* cluster is approximately 3%, whereas *jalla* and *floweri* differ by approximately 2% (Table 1). The taxa *superciliaris* and *contra* differ by approximately 1.7% (Table 1).

**TABLE 1:** Pairwise genetic distance matrix between *contra*, *superciliaris*, *floweri* and *jalla* (within the Asian Pied Starling [*Gracupica contra*] complex) and Javan Myna (*Acridotheres javanicus*) based on the mitochondrial ND2 gene

	contra				superciliaris		floweri			jalla		
*contra*												
*superciliaris*	1.6%–1.9%											
*floweri*	2.6%–3.1%				2.9%–3.2%							
*jalla*	2.8%–3.2%				3.0%–3.1%		1.7%–2.1%					
*Acridotheres javanicus*	10.3%–10.6%				9.8%		10.2%–10.4%			10.4%–10.5%		

Note

Mega7 was used to calculate raw, uncorrected pairwise distances using the pairwise deletion option for missing data.

A graph plotted between geographic distance versus genomic distance (as represented by PC2 in SNP set B2; Fig. S3b) among all geographically contiguous individuals belonging to the mainland Asian taxa illustrates that genomic variation in Asian Pied Starlings is not arranged in a geographically clinal fashion, but that there is a deep genomic division between *floweri* and *superciliaris* along a short geographic distance across the Thai–Burmese border range (Figure 2b).

Inspection of morphological traits revealed that all *floweri* individuals from Thailand for which conclusive visual material was available had a pale iris color (Table S7a). In contrast, none of the *superciliaris* individuals examined from Myanmar showed a pale iris color (Table S7a). Among individuals for which conclusive visual confirmation was possible, almost 86% of *floweri* but only 8% of *superciliaris* showed a facial morphology consistent with our definition of a large extent of bare red/orange skin around the eye (Table S7b).

## 4 DISCUSSION

### 4.1 Genomic tripartition of the Asian Pied Starling complex

Our use of genome‐wide DNA markers established three distinct evolutionary lineages within the Asian Pied Starling complex (Figure 1a, b): (a) the *contra‐superciliaris* cluster; (b) *floweri*; and (c) *jalla*. Mitochondrial DNA information corroborated this result, furnishing pairwise sequence distances between the three lineages that are within the confines of sequence divergence thresholds used for species delimitation by the general bird DNA barcoding movement (Table 1; Hajibabaei et al., 2006; Hebert et al., 2004; Johnston et al., 2011; Tizard et al., 2019), although the divergence between *jalla* (from Java) and *floweri* (from Thailand)—at ~2%—is at the lower end of divergences typically displayed by good species.

While species status for insular *jalla* from Java has been previously proposed (Birdlife International, 2020; del Hoyo et al., 2020), mainland taxa of the Asian Pied Starling are geographically contiguous across a vast range spanning ~4,000 km from easternmost Pakistan to eastern Cambodia (Figure 1a) and have never been divided into two different species in modern times. However, the deepest divergences in the complex are found between Thai *floweri* and adjacent Burmese *superciliaris* (Table 1), and our genome‐wide markers refute the hypothesis that the substantial genomic differentiation across mainland Asia is arranged in a geographic cline (Figure 2). Instead, when analyzing genomic profiles across an east–west gradient, we observe a clear spike of differentiation between samples in relatively close geographic proximity: one *superciliaris* from Bago, Myanmar, and two *floweri* samples from Bangkok, Thailand (Figure 2b). This leap in genomic differentiation across the Thai–Burmese border range (Tenasserim Hills, Dawna range, and Karen Hills) suggests a strong barrier to gene flow for these starlings (Figure 2). These mountains are a known barrier to dispersal across multiple vertebrate lineages, with recent demonstrations of deep, species‐level divergence in bats (Puechmaille et al., 2011) and bulbuls (Garg et al., 2016). In contrast, the genomic proximity of *contra* and *superciliaris* suggests that the Indo‐Burmese mountain range (Arakan mountains, Chin Hills, Naga Hills) is not an effective barrier to gene flow in these starlings (Figure 2), corroborating results from studies on other lowland vertebrates (e.g., Iyengar et al., 2005; Sadanandan et al., 2018).

### 4.2 Genomic—morphological congruence

Morphologically, the insular Javan form *jalla* has long been interpreted as the most distinct component of the Asian Pied Starling complex (e.g., Horsfield, 1821), accounting for its occasional treatment as a separate species (Birdlife International, 2020; del Hoyo et al., 2020). Our genomic result of a primary divide of Asian Pied Starlings across the Thai–Burmese border range (Figure 2a) may therefore be seen as contradicting morphological evidence. However, previous morphological inquiry has been restricted to broad qualitative assessments of plumage traits on dead museum skins. Close inspection of a collection of photographs of live individuals suggests that a previously overlooked suite of phenotypic characters may be disproportionately important in sexual advertisement and mate choice and consequently also in species delimitation across these starlings. Chief among these neglected traits is the extent of bare red facial skin and eye color. The extent of red facial skin is much larger to the east of the Thai—Burmese border range than to the west, and the eye color is invariably pale to the east of the Thai—Burmese border range but dark to the west (Figure 1c, Table S7). Crucially, such traits are not visible on dead museum skins, which only preserve the plumage of a bird but do not allow for an assessment of bare parts. Our study corroborates the importance of eye color and other bare parts as long‐neglected traits in avian species delimitation, as recently shown by another genomic analysis on South‐East Asian passerines with deep divergences across the Thai–Burmese border range (Garg et al., 2016). Ultimately, *floweri'*s closer genomic affinity with *jalla*, rather than with *superciliaris/contra* (Figure 1a,b), can easily be accounted for by the Pleistocene land bridges that have connected Java and mainland Asia during Quaternary periods of sea‐level change (Peterson et al., 2015; Voris, 2000; Whittaker & Fernández‐Palacios, 2007).

One sample from central Assam (CBT081) collected adjacent to the type locality of *sordida* in eastern Assam clustered with other individuals of *contra* in all our genomic analyses (Figure 1a,b). Due to its geographic proximity, this sample probably provides a close representation of the genomic profile of the unsampled subspecies *sordida*. Noting the dubious morphological distinction of *sordida* from the nominate *contra* and discrepancies regarding the exact circumscription of its distribution (Ripley, 1950), our genomic results (Figure 1a,b) are consistent with a synonymization of *sordida* with *contra* in future taxonomic classifications.

In summary, we propose a separation of the complex into the following three species: (a) *Gracupica contra* (Indian Pied Starling), with subspecies *contra* (most of the Indian subcontinent, including a synonymized *sordida* from eastern Assam) and *superciliaris* (mostly Myanmar and adjacent Yunnan, with a southeastern range boundary here confirmed to include southern Myanmar); (b) *Gracupica floweri* (Thai Pied Starling), monotypic (mostly Thailand and Cambodia); and (c) *Gracupica jalla* (Javan Pied Starling), monotypic (historically Java and Bali, now extinct in the wild).

### 4.3 Use of historical DNA in the conservation of endangered taxa affected by introgression

Conservation management often hinges upon clear taxonomic resolution (Ely et al., 2017; McCarthy, 2014; Regan et al., 2002). In many terminally endangered organisms, taxonomic research is impaired by the rarity of the species involved (Tsai et al., 2019) exacerbated by potential genomic admixture affecting the last surviving populations (Pilot et al., 2019). Human‐mediated hybridization, either in captivity or in the wild via habitat shifts (Mallet et al., 2011), invasions by alien species (Bleeker et al., 2007), wildlife trade (Nijman et al., 2018), and accidental release (Baveja et al., 2019), among other factors, may compromise the genetic integrity of the last survivors of a terminally endangered population and confound taxonomic research. For instance, one of our study lineages, the Javan Pied Starling (*G. jalla*), is heavily traded in Indonesian markets and frequently admixed with *floweri* and *superciliaris* in illegal captivity due to a lack of recruitment of new individuals resulting from their extinction in the wild. Our study was only possible because of the availability of museum samples from a time preceding such introgression.

### 4.4 Conservation recommendations

The captive *jalla* individuals from Bali Bird Park emerged with an identical genomic profile to historical *jalla* samples, indicating a fortunate lack of admixture (Figure 1a). They are therefore suitable founding individuals for a conservation breeding program of Javan Pied Starlings, which—at the time of writing—does not yet exist. In 2018, the Ministry of Environment and Forestry of Indonesia removed Javan Pied Starlings (local name: *jalak suren*) and four other species of songbirds from the list of protected species because of advocacy work by the bird‐keepers’ lobby (Jakarta Hidayat, 2020; Post, 2018). Despite the Javan Pied Starling's extinction in the wild, the previous lack of a scientific consensus regarding its taxonomic status has probably contributed to hesitations in affording this taxon full protection. Urgent conservation attention is hence imperative to prevent the species from going extinct even in captivity, for example, through rampant cross‐breeding with *G. floweri*.

At a time when the survival of more and more species hinges on conservation breeding (Chattopadhyay et al., 2019; Çilingir et al., 2017; Galla et al., 2020; Wright et al., 2020), our study is a unique demonstration of the successful use of historical DNA material to derive genome‐wide DNA marker sets that clarify the taxonomic status of a species that is extinct in the wild and increasingly admixed in captivity. Historical DNA is a potentially underutilized resource (Bi et al., 2013; Payne & Sorenson, 2002; Shaffer et al., 1998; Suarez & Tsutsui, 2004). In conservation problems such as the pied starlings of Asia, a definitive conservation strategy would likely be impossible to design without the clear evidence provided by recourse to historical DNA. We encourage a broader utilization of historical samples using the laboratory and bioinformatic methods implemented in our study for a lasting resolution of controversial dilemmas in conservation involving terminally endangered species.

## 5 DATA ARCHIVING STATEMENT

The raw sequence data for this study are available at Sequence Read Archive, NCBI under BioProject PRJNA669910. Additional details and results of this study are available in the supporting information of this article.

## Supporting information

Supplementary Material
