# Supplementary material for: Using historical genome‐wide DNA to unravel the confused taxonomy in a songbird lineage that is extinct in the wild
Source: Evol Appl. 2020 Nov 7;14(3):698–709. doi: 10.1111/eva.13149 (PMC7980273; doi:10.1111/eva.13149)
Supplement: Supplementary file 1 — Supplementary Material [file EVA-14-698-s001.docx]

**Supporting Information**

**TABLES**

Table S1 Details of historical and contemporary tissue samples.

| **Sample name** | **Sample Code** | **Holding Institution** | **Collection locality** | **Taxon name** | **Sample type** | **Collection date** |
| --- | --- | --- | --- | --- | --- | --- |
| CBT077_contra* | SKIN 63569 | AMNH | Agra, India | *contra* | skin | June 1892 |
| CBT076_jalla* | SKIN 666626 | AMNH | Java, Indonesia | *jalla* | skin | October 1897 to January 1898 |
| CBT078_superciliaris* | ROM Birds 49368 | ROM | Bago (Lower Bago River), Burma | *superciliaris* | skin | 5 April 1878 |
| CBT080_contra* | SKIN 178499 | AMNH | Nepal | *contra* | skin | 10 March 1923 |
| CBT084_jalla* | SKIN 666622 | AMNH | East Java, Indonesia | *jalla* | skin | 17 November 1927 |
| CBT082_floweri* | SKIN 666618 | AMNH | Bangkok, Thailand | *floweri* | skin | 9 February 1915 |
| CBT083_floweri* | SKIN 265921 | AMNH | Bangkok, Thailand | *floweri* | skin | 11 March 1927 |
| CBT086_contra* | ROM Birds 147441 | ROM | Meerut, Uttar Pradesh, India | *contra* | skin | 27 January 1983 |

Table S1 (continued).

| **Sample name** | **Sample Code** | **Holding Institution** | **Collection locality** | **Taxon name** | **Sample type** | **Collection date** |
| --- | --- | --- | --- | --- | --- | --- |
| CBT079_contra* | ROM Birds 136434 | ROM | Lucknow, Uttar Pradesh, India | *contra* | skin | 18 November 1979 |
| CBT085_superciliaris* | UMMZ Birds 178064 | UMMZ | Manipur (Kanglatongbi), India | *superciliaris* | skin | 30 December 1950 |
| CBT075_contra* | UMMZ Birds 178057 | UMMZ | Assam (Kamrup: Palasbari), India | *contra* | skin | 10 June 1953 |
| CBT081_contra* | UMMZ Birds 178058 | UMMZ | Assam (Kamrup: Palasbari), India | *contra* | skin | 12 June 1953 |
| BBPS01_jalla | _ | Bali Bird Park | _ | *jalla* | blood | _ |
| BBPS02_jalla | _ | Bali Bird Park | _ | *jalla* | blood | _ |
| BBPS03_jalla | _ | Bali Bird Park | _ | *jalla* | blood | _ |
| JBPS04_jalla | _ | Jurong Bird Park | _ | *jalla* | blood | _ |
| BMS05_jalla | _ | Bali Bird Park | _ | *jalla* | blood | _ |
| BMS06_jalla | _ | Bali Bird Park | _ | *jalla* | blood | _ |
| BMS07_jalla | _ | Bali Bird Park | _ | *jalla* | blood | _ |
| BMS08_jalla | _ | Bali Bird Park | _ | *jalla* | blood | _ |
| JBPS01_contra | _ | Jurong Bird Park | _ | *contra* | blood | _ |

Asterisk (*) indicates historical sample obtained from museums. The sample code is each specimen’s ID at its respective museum. Abbreviations: AMNH- American Museum of Natural History (New York), UMMZ- University of Michigan Museum of Zoology (Ann Arbor), ROM- Royal Ontario Museum (Toronto).

Table S2 Summary of efficiency of target enrichment and locus recovery using the perl script *hybpiper_stats.py* in HybPiper v1.3.1. Sample names refer to Table S1.

| **Sample name** | **NumReads** | **ReadsMapped** | **%onTarget** | **GenesWithSeqs** | **% loci recovered** |
| --- | --- | --- | --- | --- | --- |
|  |  |  |  |  |  |
| CBT080_contra | 29132146 | 10317020 | 35.4 | 978 | 99.8 |
| BMS08_jalla | 6364255 | 3946884 | 62 | 977 | 99.69 |
| CBT077_contra | 20179107 | 6180378 | 30.6 | 977 | 99.69 |
| CBT086_contra | 26731169 | 8106754 | 30.3 | 979 | 99.9 |
| CBT081_contra | 16478181 | 1727155 | 10.5 | 976 | 99.59 |
| CBT079_contra | 42736112 | 12926405 | 30.2 | 979 | 99.9 |
| JBPS01_contra | 11328860 | 6584712 | 58.1 | 979 | 99.9 |
| CBT078_superciliaris | 17576166 | 10199782 | 58 | 979 | 99.9 |
| CBT076_jalla | 15817852 | 1818121 | 11.5 | 968 | 98.78 |
| CBT082_floweri | 42648121 | 16262903 | 38.1 | 979 | 99.9 |
| CBT083_floweri | 27821567 | 13027505 | 46.8 | 979 | 99.9 |
| CBT075_contra | 1067563 | 256771 | 24.1 | 225 | 22.96 |
| CBT084_jalla | 29599087 | 15457884 | 52.2 | 979 | 99.9 |
| BBPS01_jalla | 7177725 | 5308607 | 74 | 979 | 99.9 |
| BBPS02_jalla | 7112293 | 5100031 | 71.7 | 978 | 99.8 |
| BBPS03_jalla | 10477726 | 7734463 | 73.8 | 979 | 99.9 |
| JBPS04_jalla | 5541847 | 4035836 | 72.8 | 978 | 99.8 |
| BMS05_jalla | 2917487 | 2035953 | 69.8 | 978 | 99.8 |
| BMS06_jalla | 3699814 | 2182402 | 59 | 978 | 99.8 |
| BMS07_jalla | 5290885 | 3132175 | 59.2 | 978 | 99.8 |
| CBT085_superciliaris | 33467240 | 11985645 | 35.8 | 979 | 99.9 |

NumReads is the total number of reads for each sample. ReadsMapped is the number of reads that mapped to target. %onTarget is the percentage of reads that mapped to target. GeneswithSeq is the number of loci for which sequences were retrieved. % loci recovered is the percentage of loci for which sequences were retrieved.

Table S3 Details of the selected candidate sequence loci with >20 SNPs per locus.

| **Locus identity** | **SNP count** | **Gene name** | **Gene function** | **Best model (AICc)** |
| --- | --- | --- | --- | --- |
| locus038 | 23 | ZNF804B | unknown | TIM1+I |
| locus043 | 37 | SEL1L3 | transmembrane protein involved in cell proliferation | TIM2+I+G |
| locus044 | 22 | NWD2 | pathogen recognition receptor | TPM1uf+I |
| locus089 | 23 | DOK7 | formation of neuromuscular synapse | TVM+G |
| locus130 | 24 | CASP8AP2 | apoptosis & glucocorticoid secretion | HKY+I |
| locus131 | 23 | ZNF292 | tumor suppressor locus | locus not retrieved |
| locus179 | 22 | IFIT5/LOC100229421 | unknown | GTR+I |
| locus186 | 21 | PTPN12 | tumor suppressor, protects against oxidative stress | TVM+G |
| locus203 | 21 | ANKS1B | post-synaptic signaling, post-natal development | TIM2+I |
| locus205 | 21 | BRCA2 | tumor suppressor, DNA repair | TrN+I |
| locus330 | 29 | CENPF | chromosome segregation | TVM+G |
| locus358 | 21 | SPATA13 | cell migration, social hierarchy, nocturnal activity | TPM1uf+G |
| locus419 | 23 | ABCA13 | transporter | TVM+G |
| locus420 | 41 | SETX | DNA & RNA processing | TIM3+I |
| locus528 | 47 | TRIM25 | innate response to infection | TIM2+I |
| locus547 | 27 | URB2 | ribosome biogenesis | TIM1+I |
| locus563 | 35 | FAT4 | cell polarity & tumor suppressor | TrN+I+G |
| locus621 | 46 | MFSD6 | transportation across biomembranes | TPM2uf+I |
| locus658 | 56 | MK167 | cellular proliferation | HKY+G |
| locus713 | 24 | FMN2 | actin cytoskeleton, cell polarity & DNA damage, protein transport, stress response | TIM1+I |
| locus771 | 26 | LOC100222288 | unknown | TIM1+I+G |

Table S3 (continued).

| **Locus identity** | **SNP count** | **Gene name** | **Gene function** | **Best model (AICc)** |
| --- | --- | --- | --- | --- |
| locus784 | 35 | DCHS2/LOC100219721 | cell adhesion protein/craniofacial development | TPM3uf+I |
| locus799 | 23 | ETAA1 | cope replication stress | TPM1uf+G |
| locus979 | 41 | TAS1R3 | taste response | SYM+I+G |
| locus982 | 38 | CZH5orf30/MACIR | inflammatory/immune response | TPM3uf+I |
| ND2 | _ | NADH dehydrogenase 2 | NADH dehydrogenation | TIM1+G |

SNP count is the number of SNPs for each locus. Gene name was retrieved by performing BLAST between the target locus sequence and the Zebra Finch genome (*Taeniopygia guttata*, GCF_003957565.1) available on NCBI. Gene function was obtained via a Google search. Best model indicates the best nucleotide substitution model for each locus based on lowest corrected Akaike information criterion (AICc) after performing jModelTest v.2.1.10.

Table S4 Example of mapDamage output for one historical sample, CBT083_floweri (see Table S1), indicating frequencies of cytosine to thymine mutations per position from the 5' ends and guanine to adenine mutations per position from the 3' ends.

| **Position** | **3pG>A** | **5pC>T** |
| --- | --- | --- |
| 1 | 0.01134455 | 0.0099864 |
| 2 | 0.01028077 | 0.01003847 |
| 3 | 0.00694192 | 0.00693084 |
| 4 | 0.00726977 | 0.00727856 |
| 5 | 0.00725696 | 0.00741163 |
| 6 | 0.00801169 | 0.00792443 |
| 7 | 0.00786978 | 0.00786959 |
| 8 | 0.00801968 | 0.00807612 |
| 9 | 0.00814937 | 0.00804066 |
| 10 | 0.00775162 | 0.00761923 |
| 11 | 0.00775764 | 0.00763802 |
| 12 | 0.00764639 | 0.00753387 |
| 13 | 0.00739474 | 0.00727747 |
| 14 | 0.00756747 | 0.00750497 |
| 15 | 0.00792074 | 0.00783732 |
| 16 | 0.00765147 | 0.00767612 |
| 17 | 0.00780524 | 0.00777147 |
| 18 | 0.00743204 | 0.00742896 |
| 19 | 0.00788377 | 0.00774029 |
| 20 | 0.00786805 | 0.0078763 |
| 21 | 0.00806618 | 0.00799725 |
| 22 | 0.00786833 | 0.00784839 |
| 23 | 0.00788716 | 0.0077924 |
| 24 | 0.00779013 | 0.00764127 |
| 25 | 0.00769983 | 0.00760166 |

Table S5 Results of gene ontology (GO) enrichment tests in Blast2Go for 25 selected candidate loci, each with >20 SNPs per locus, using a corrected p-value by false discovery rate (FDR) of 0.05 against a reference set of 980 loci.

| **Tags** | **GO ID** | **GO Name** | **GO Category** | **FDR** | **P-Value** |
| --- | --- | --- | --- | --- | --- |
| [OVER] | GO:0098742 | cell-cell adhesion via plasma-membrane adhesion molecules | BIOLOGICAL_PROCESS | 1 | 0.00875511 |
| [UNDER] | GO:0016043 | cellular component organization | BIOLOGICAL_PROCESS | 1 | 0.03800539 |
| [OVER] | GO:0004850 | uridine phosphorylase activity | MOLECULAR_FUNCTION | 1 | 0.02543235 |
| [OVER] | GO:1903767 | sweet taste receptor complex | CELLULAR_COMPONENT | 1 | 0.02543235 |
| [OVER] | GO:1903768 | taste receptor complex | CELLULAR_COMPONENT | 1 | 0.02543235 |
| [OVER] | GO:0008527 | taste receptor activity | MOLECULAR_FUNCTION | 1 | 0.02543235 |
| [OVER] | GO:0010764 | negative regulation of fibroblast migration | BIOLOGICAL_PROCESS | 1 | 0.02543235 |
| [OVER] | GO:0010761 | fibroblast migration | BIOLOGICAL_PROCESS | 1 | 0.02543235 |
| [UNDER] | GO:0071704 | organic substance metabolic process | BIOLOGICAL_PROCESS | 1 | 0.02920482 |
| [OVER] | GO:0010762 | regulation of fibroblast migration | BIOLOGICAL_PROCESS | 1 | 0.02543235 |
| [OVER] | GO:0007156 | homophilic cell adhesion via plasma membrane adhesion molecules | BIOLOGICAL_PROCESS | 1 | 0.00592907 |
| [UNDER] | GO:0008152 | metabolic process | BIOLOGICAL_PROCESS | 1 | 0.0108448 |
| [UNDER] | GO:0010467 | gene expression | BIOLOGICAL_PROCESS | 1 | 0.03810731 |
| [OVER] | GO:0001582 | detection of chemical stimulus involved in sensory perception of sweet taste | BIOLOGICAL_PROCESS | 1 | 0.02543235 |
| [OVER] | GO:0002102 | podosome | CELLULAR_COMPONENT | 1 | 0.02543235 |
| [OVER] | GO:0006222 | UMP biosynthetic process | BIOLOGICAL_PROCESS | 1 | 0.02543235 |
| [OVER] | GO:0006220 | pyrimidine nucleotide metabolic process | BIOLOGICAL_PROCESS | 1 | 0.02543235 |

Table S5 (continued).

| **Tags** | **GO ID** | **GO Name** | **GO Category** | **FDR** | **P-Value** |
| --- | --- | --- | --- | --- | --- |
| [OVER] | GO:0006221 | pyrimidine nucleotide biosynthetic process | BIOLOGICAL_PROCESS | 1 | 0.02543235 |
| [UNDER] | GO:0090304 | nucleic acid metabolic process | BIOLOGICAL_PROCESS | 1 | 0.02352613 |
| [OVER] | GO:0009218 | pyrimidine ribonucleotide metabolic process | BIOLOGICAL_PROCESS | 1 | 0.02543235 |
| [OVER] | GO:0009220 | pyrimidine ribonucleotide biosynthetic process | BIOLOGICAL_PROCESS | 1 | 0.02543235 |
| [OVER] | GO:0072528 | pyrimidine-containing compound biosynthetic process | BIOLOGICAL_PROCESS | 1 | 0.02543235 |
| [UNDER] | GO:0043170 | macromolecule metabolic process | BIOLOGICAL_PROCESS | 1 | 0.01374907 |
| [OVER] | GO:0043173 | nucleotide salvage | BIOLOGICAL_PROCESS | 1 | 0.02543235 |
| [UNDER] | GO:0043227 | membrane-bounded organelle | CELLULAR_COMPONENT | 1 | 0.0373281 |
| [OVER] | GO:0033041 | sweet taste receptor activity | MOLECULAR_FUNCTION | 1 | 0.02543235 |
| [OVER] | GO:0046049 | UMP metabolic process | BIOLOGICAL_PROCESS | 1 | 0.02543235 |
| [OVER] | GO:0016235 | aggresome | CELLULAR_COMPONENT | 1 | 0.02543235 |
| [UNDER] | GO:0009987 | cellular process | BIOLOGICAL_PROCESS | 1 | 0.00834394 |
| [OVER] | GO:0008655 | pyrimidine-containing compound salvage | BIOLOGICAL_PROCESS | 1 | 0.02543235 |
| [OVER] | GO:0044206 | UMP salvage | BIOLOGICAL_PROCESS | 1 | 0.02543235 |
| [OVER] | GO:0050909 | sensory perception of taste | BIOLOGICAL_PROCESS | 1 | 0.02543235 |
| [OVER] | GO:0032262 | pyrimidine nucleotide salvage | BIOLOGICAL_PROCESS | 1 | 0.02543235 |
| [OVER] | GO:0050917 | sensory perception of umami taste | BIOLOGICAL_PROCESS | 1 | 0.02543235 |
| [OVER] | GO:0050916 | sensory perception of sweet taste | BIOLOGICAL_PROCESS | 1 | 0.02543235 |
| [UNDER] | GO:0044238 | primary metabolic process | BIOLOGICAL_PROCESS | 1 | 0.02959673 |
| [UNDER] | GO:0044237 | cellular metabolic process | BIOLOGICAL_PROCESS | 1 | 0.04689493 |

Table S5 (continued).

| **Tags** | **GO ID** | **GO Name** | **GO Category** | **FDR** | **P-Value** |
| --- | --- | --- | --- | --- | --- |
| [OVER] | GO:0050912 | detection of chemical stimulus involved in sensory perception of taste | BIOLOGICAL_PROCESS | 1 | 0.02543235 |
| [UNDER] | GO:0006807 | nitrogen compound metabolic process | BIOLOGICAL_PROCESS | 1 | 0.04654523 |
| [UNDER] | GO:0071840 | cellular component organization or biogenesis | BIOLOGICAL_PROCESS | 1 | 0.0382497 |
| [UNDER] | GO:0009059 | macromolecule biosynthetic process | BIOLOGICAL_PROCESS | 1 | 0.03794336 |
| [UNDER] | GO:0044260 | cellular macromolecule metabolic process | BIOLOGICAL_PROCESS | 1 | 0.0373281 |
| [OVER] | GO:0009123 | nucleoside monophosphate metabolic process | BIOLOGICAL_PROCESS | 1 | 0.02543235 |
| [OVER] | GO:0009124 | nucleoside monophosphate biosynthetic process | BIOLOGICAL_PROCESS | 1 | 0.02543235 |
| [OVER] | GO:0009129 | pyrimidine nucleoside monophosphate metabolic process | BIOLOGICAL_PROCESS | 1 | 0.02543235 |
| [OVER] | GO:0009156 | ribonucleoside monophosphate biosynthetic process | BIOLOGICAL_PROCESS | 1 | 0.02543235 |
| [OVER] | GO:0010138 | pyrimidine ribonucleotide salvage | BIOLOGICAL_PROCESS | 1 | 0.02543235 |
| [OVER] | GO:0009161 | ribonucleoside monophosphate metabolic process | BIOLOGICAL_PROCESS | 1 | 0.02543235 |
| [OVER] | GO:0009130 | pyrimidine nucleoside monophosphate biosynthetic process | BIOLOGICAL_PROCESS | 1 | 0.02543235 |
|  |  |  |  |  |  |

Table S5 (continued).

| **Tags** | **GO ID** | **GO Name** | **GO Category** | **FDR** | **P-Value** |
| --- | --- | --- | --- | --- | --- |
| [OVER] | GO:0043094 | cellular metabolic compound salvage | BIOLOGICAL_PROCESS | 1 | 0.02543235 |
| [OVER] | GO:0009174 | pyrimidine ribonucleoside monophosphate biosynthetic process | BIOLOGICAL_PROCESS | 1 | 0.02543235 |
| [OVER] | GO:0009173 | pyrimidine ribonucleoside monophosphate metabolic process | BIOLOGICAL_PROCESS | 1 | 0.02543235 |

Tags 'OVER' and 'UNDER' represent GO IDs that are over - or under-represented in the selected loci.

Table S6 Results of over-representation analysis (ORA) for the 25 selected candidate loci with >20 SNPs per locus against a reference set of 980 loci using the web-based tool WebGestalt to show the 10 most over-represented gene ontology (GO) IDs.

| **GO ID** | **Description** | **Size** | **Expect** | **Ratio** | **P Value** | **FDR** |
| --- | --- | --- | --- | --- | --- | --- |
| GO:0042802 | identical protein binding | 18 | 0.38462 | 10.4 | 0.00028 | 0.33722 |
| GO:0031297 | replication fork processing | 2 | 0.04274 | 46.8 | 0.00041 | 0.33722 |
| GO:0022613 | ribonucleoprotein complex biogenesis | 9 | 0.19231 | 15.6 | 0.00055 | 0.33722 |
| GO:0000228 | nuclear chromosome | 9 | 0.19231 | 15.6 | 0.00055 | 0.33722 |
| GO:0005694 | chromosome | 24 | 0.51282 | 7.8 | 0.00092 | 0.37126 |
| GO:0045005 | DNA-dependent DNA replication maintenance of fidelity | 3 | 0.0641 | 31.2 | 0.00122 | 0.37126 |
| GO:0008022 | protein C-terminus binding | 3 | 0.0641 | 31.2 | 0.00122 | 0.37126 |
| GO:0002020 | protease binding | 3 | 0.0641 | 31.2 | 0.00122 | 0.37126 |
| GO:0000793 | condensed chromosome | 4 | 0.08547 | 23.4 | 0.00241 | 0.58721 |
| GO:0030496 | midbody | 4 | 0.08547 | 23.4 | 0.00241 | 0.58721 |

Table S7 Results of morphological trait inspection of *floweri* from Thailand and *superciliaris* from Myanmar in the Asian Pied Starling complex.

(a) Presence of pale eye color

| Taxon examined | Oriental Bird Images | | | | | eBird | | | |
| --- | --- | --- | --- | --- | --- | --- | --- | --- | --- |
|  | Total number of birds examined (n) | Present | | Absent | Indeterminate | Total number of birds examined (n) | Present | Absent | Indeterminate |
| *floweri* | 13 | | 12 | 0 | 1 | 318 | 278 | 0 | 40 |
| *superciliaris* | 1 | | 0 | 1 | 0 | 23 | 0 | 13 | 10 |

Pictures were sourced from Oriental Bird Images (www.orientalbirdimages.org) and eBird (www.ebird.org). Traits were recorded as ‘Indeterminate’ in photos of insufficient quality or those in which the bird was too distant or depicted at an unsuitable angle.

**(b) Presence of large bare facial skin**

| Taxon examined | Oriental Bird Images | | | | eBird | | | |
| --- | --- | --- | --- | --- | --- | --- | --- | --- |
|  | Total number of birds examined (n) | Present | Absent | Indeterminate | Total number of birds examined (n) | Present | Absent | Indeterminate |
| *floweri* | 13 | 8 | 4 | 1 | 318 | 180 | 27 | 111 |
| *superciliaris* | 1 | 0 | 1 | 0 | 23 | 1 | 11 | 11 |

Pictures were sourced from Oriental Bird Images (www.orientalbirdimages.org) and eBird (www.ebird.org). If the length of bare facial skin extending from the posterior end of eye towards the nape was greater than the length of bare skin extending from anterior end of the eye to the base of the bill, the bare facial skin was considered 'large'. Traits were recorded as ‘Indeterminate’ in photos of insufficient quality or those in which the bird was too distant or depicted at an unsuitable angle.

**FIGURES**

| 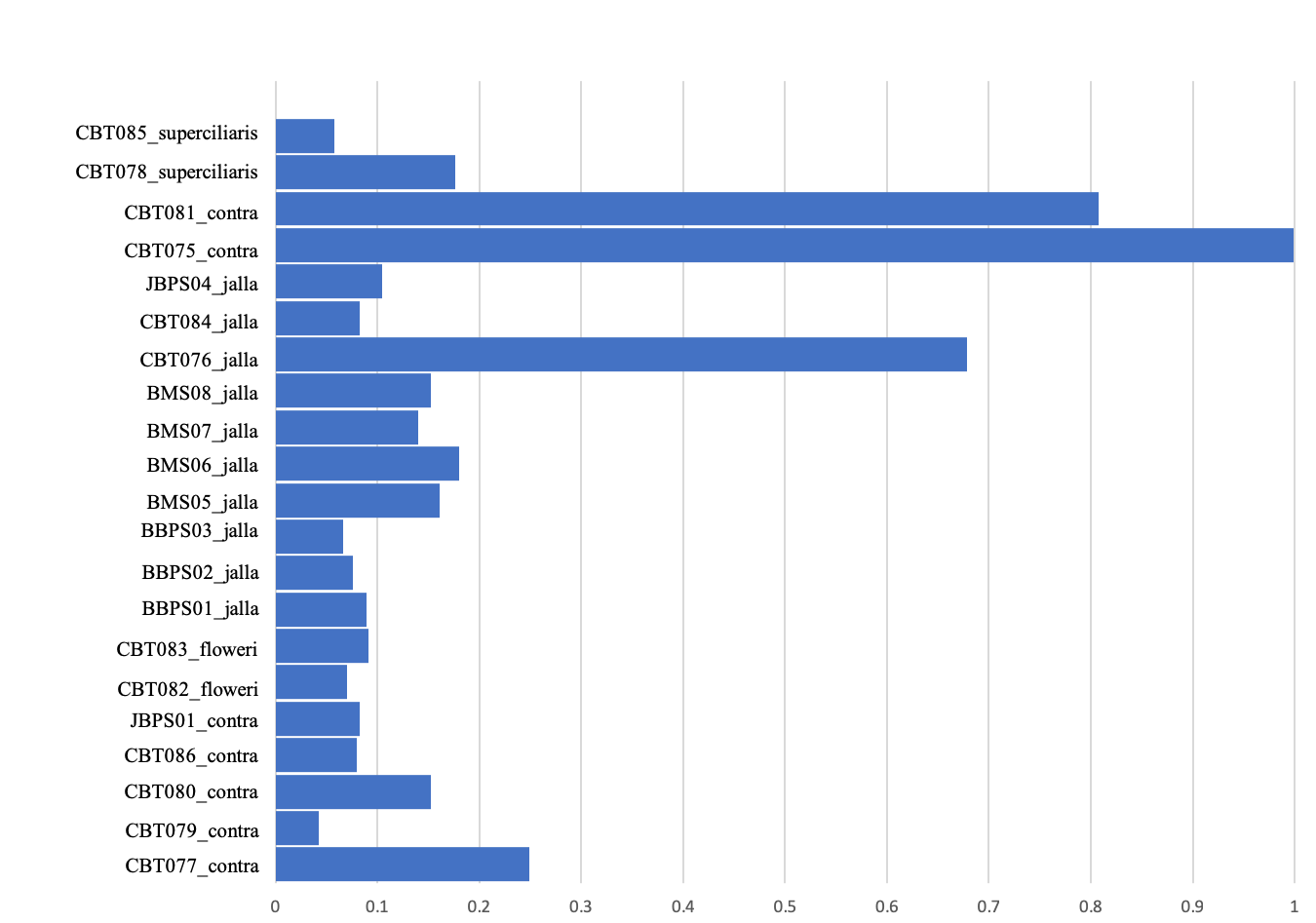 |
| --- |

Figure S1 Bar graph showing % missingness in the variant file of each individual sample resulting from the SNP calling pipeline.

The y-axis represents individual samples (Table S1) and the x-axis represents the fraction of missingness in each sample.

| 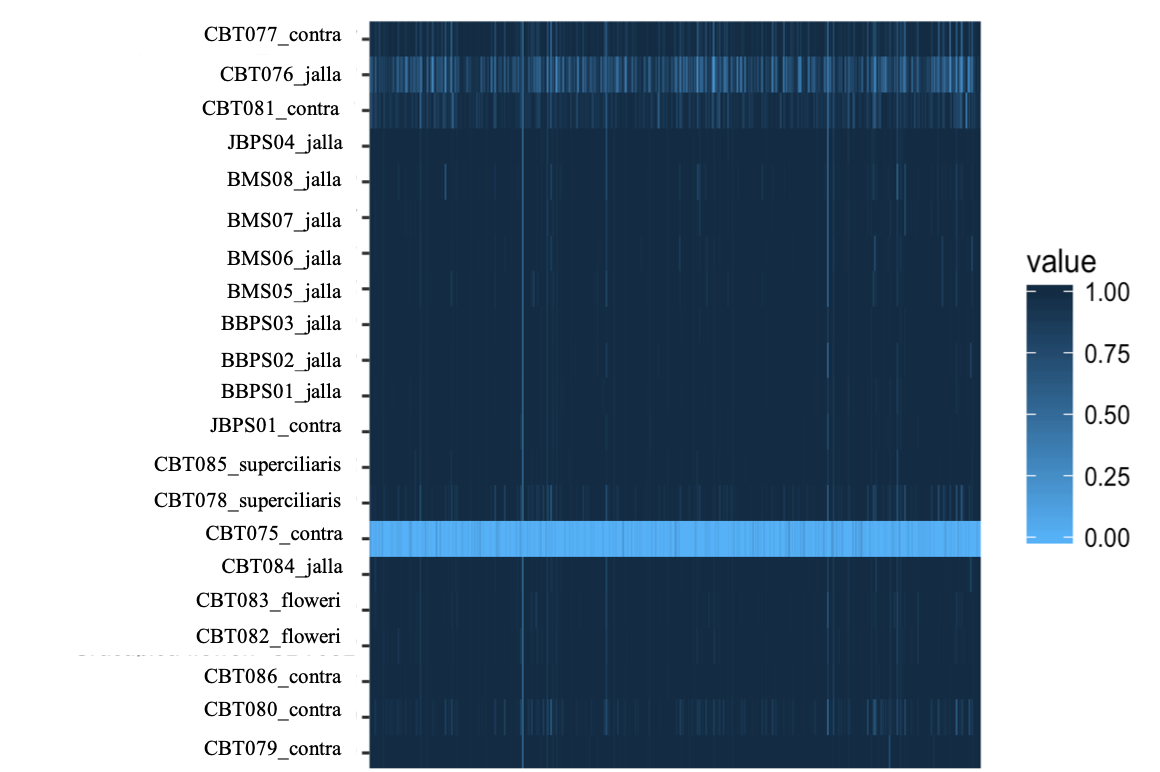 |
| --- |

**Figure S2 Heat map showing efficiency of locus recovery for the 980 loci that were enriched by in-solution hybridization.**

Each row represents a single sample and each column represents a single locus. Shades of blue indicate the fraction of length of each recovered locus.

| 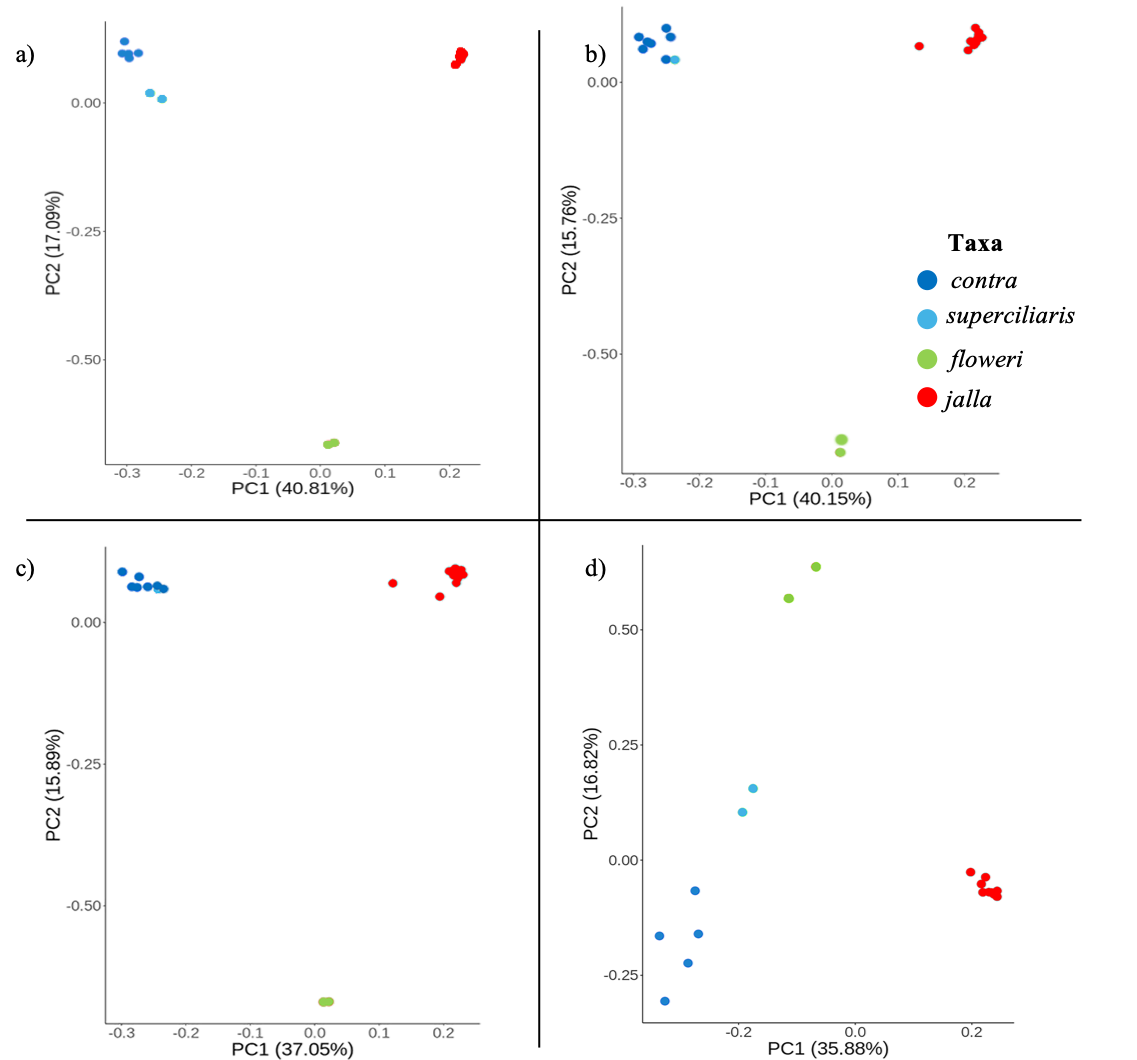 |
| --- |

Figure S3 Principal component analysis (PCA) plot for Asian Pied Starlings (*Gracupica contra*).

The PCA is based on (a) a linked variant set of 6333 SNPs (SNP set A1), (b) an unlinked variant set of 412 SNPs (SNP set B2), (c) a linked variant set of 871 SNPs (SNP set B1), and (d) a linked variant set of 749 SNPs from 25 selected loci (each with >20 SNPs per locus; SNP set C). Each colored dot represents a single individual and the color depicts the taxon.

| 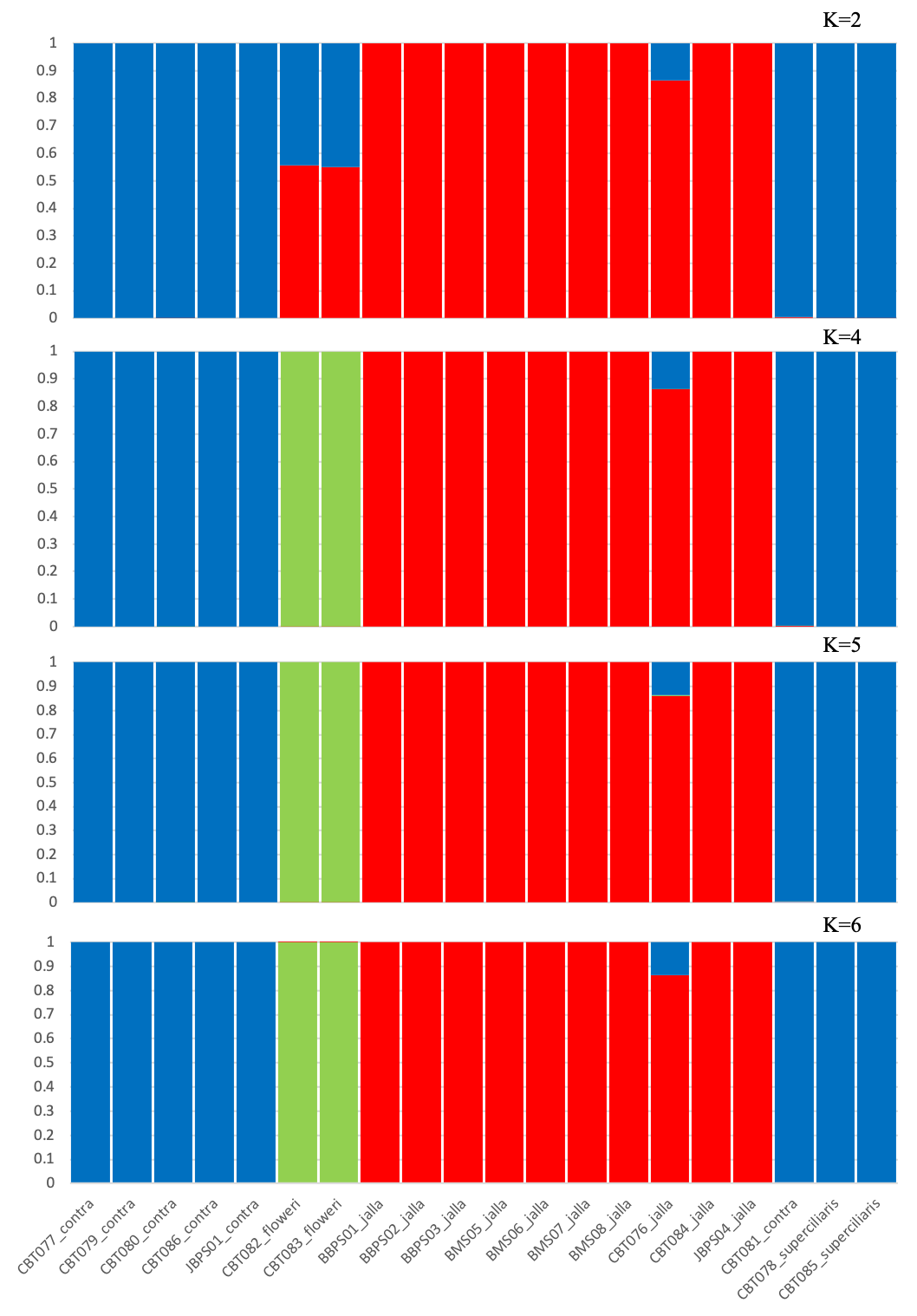 |
| --- |

Figure S4 (a) Population structure of the Asian Pied Starling (*Gracupica contra*) complex based on 412 unlinked genome-wide SNPs (SNP set B2) obtained using STRUCTURE at K= 2, 4, 5 and 6.

Each stacked column represents a single individual. Each color represents a different ancestral contribution. The y-axis represents the fraction of ancestral contributions.

| 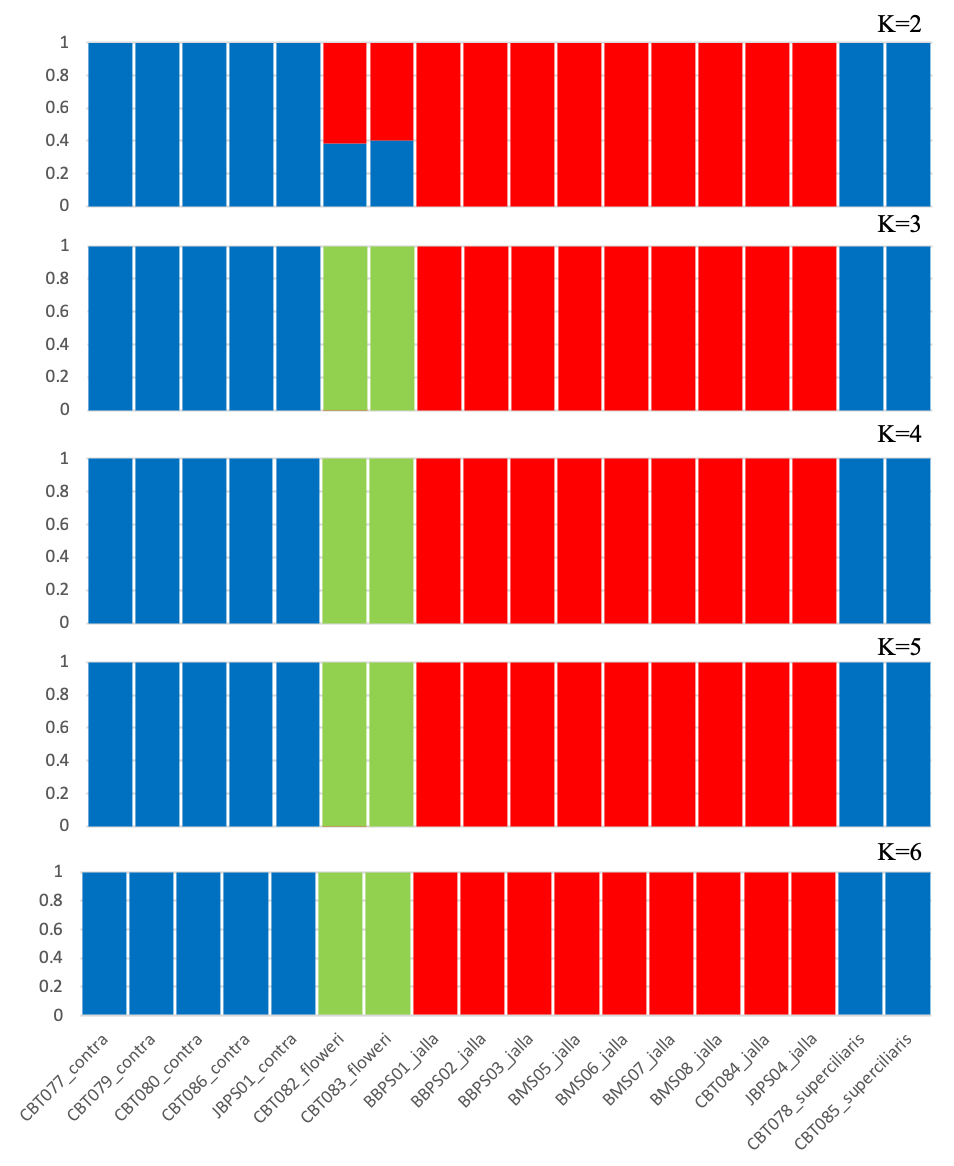 |
| --- |

Figure S4 (b) Population structure of the Asian Pied Starling (*Gracupica contra*) complex based on 936 unlinked genome-wide SNPs (SNP set A2) obtained using STRUCTURE at K=2-6.

Each stacked column represents a single individual. Each color represents a different ancestral contribution. The y-axis represents the fraction of ancestral contributions.

(a) locus 038

| 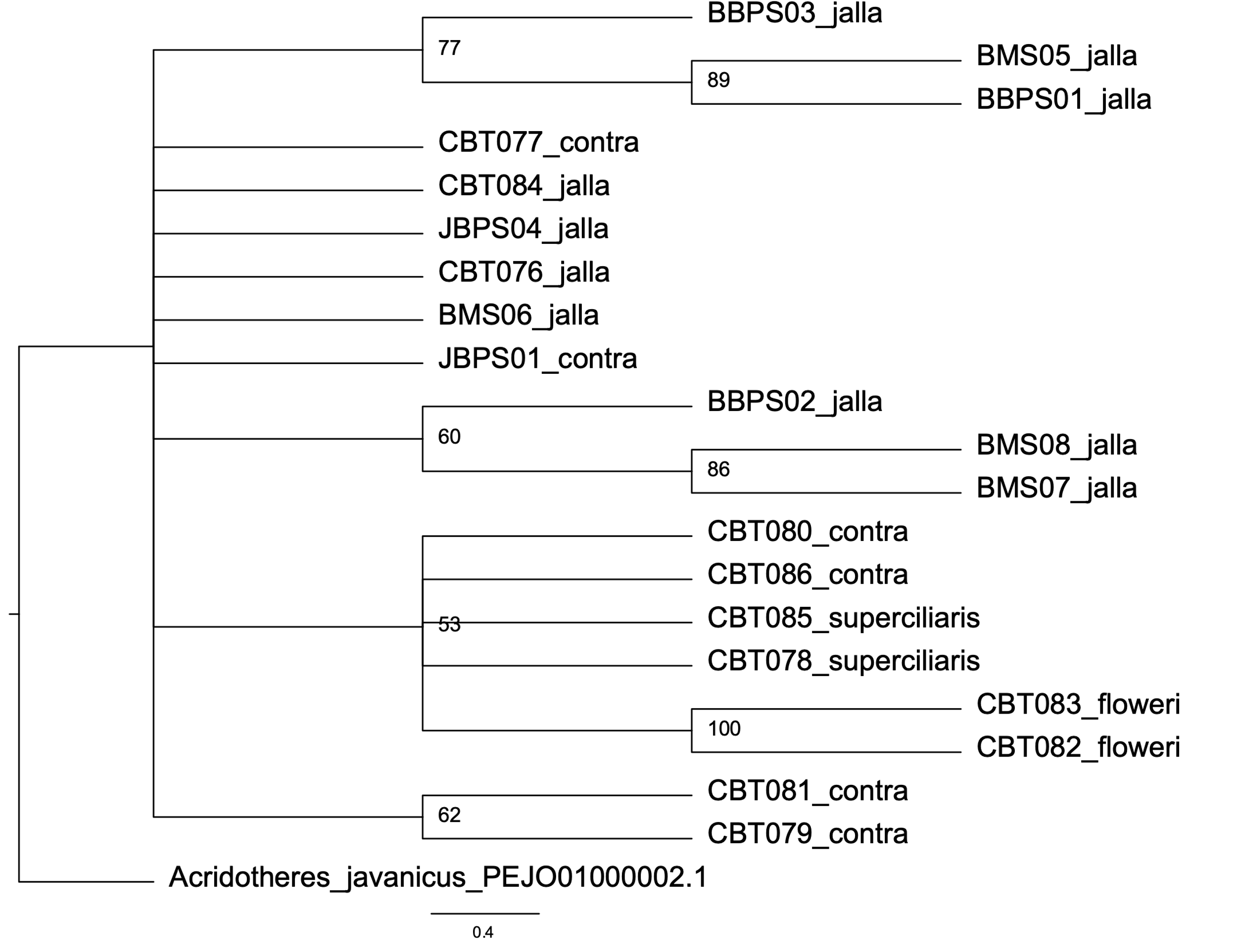 |
| --- |

Figure S5 (a-y) Maximum Likelihood consensus trees based on 24 selected candidate loci (each with >20 SNPs per locus) with bootstrap support ≥ 50% shown beside nodes.

(b) locus 043

| 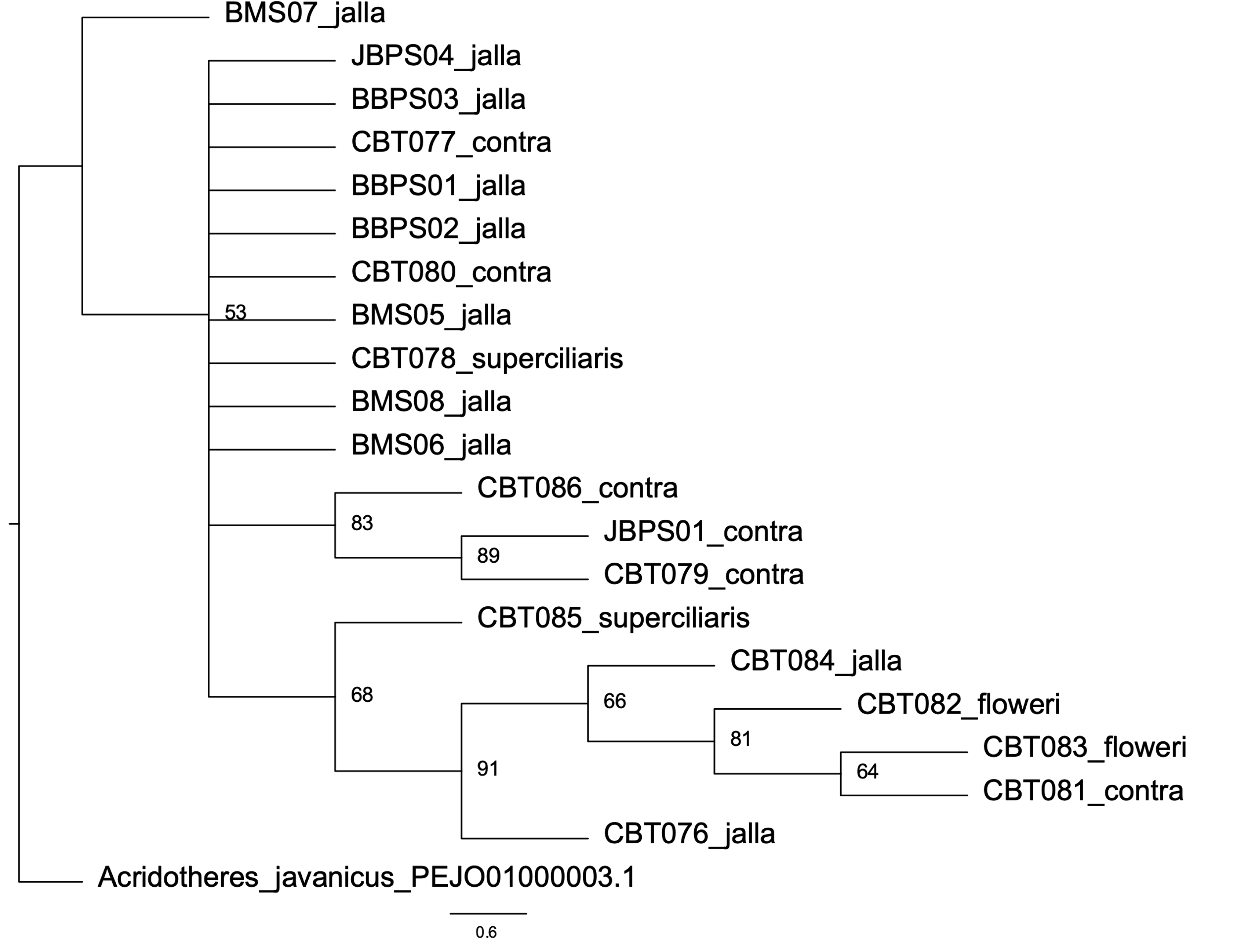 |
| --- |

Figure S5 (continued).

(c) locus 044

| 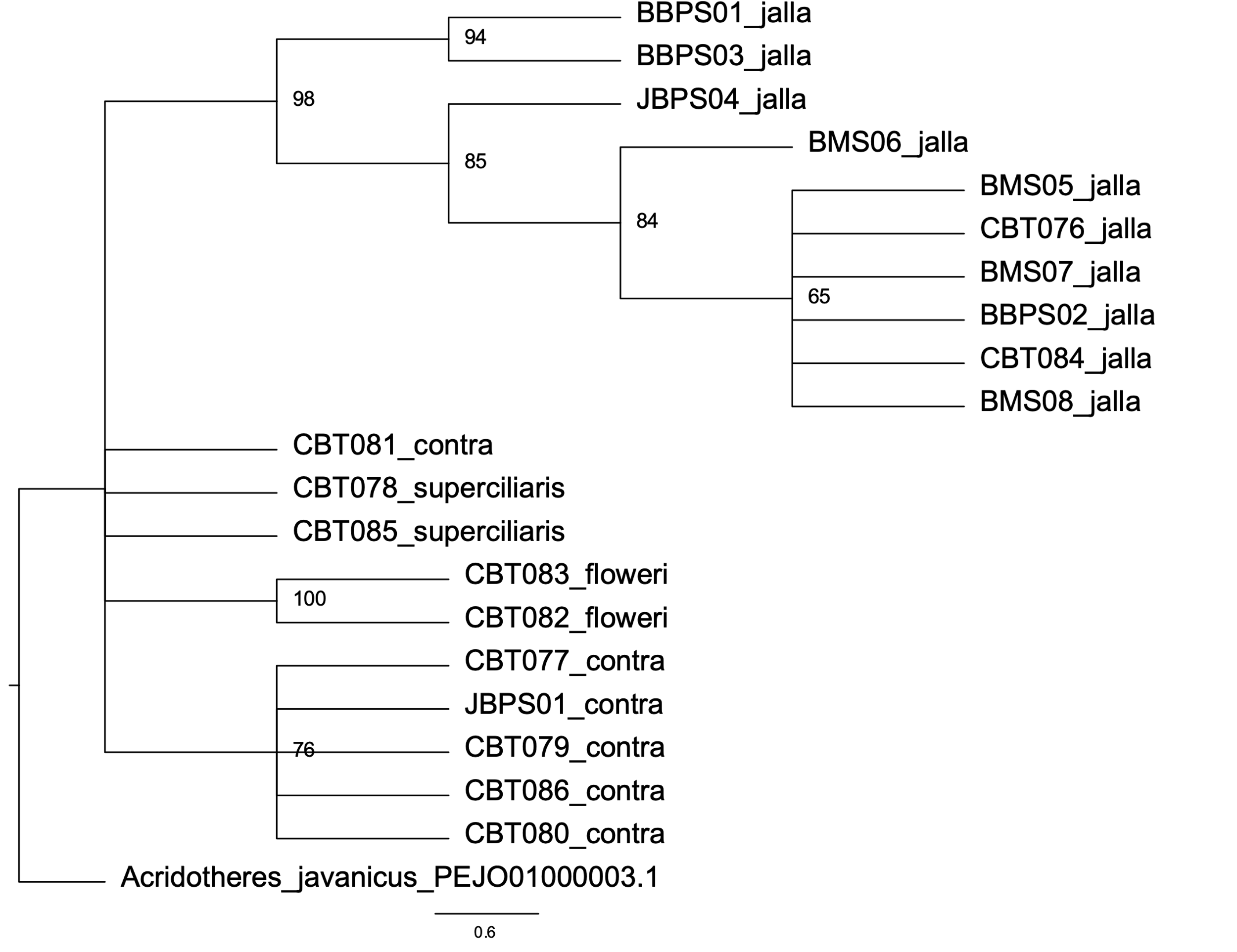 |
| --- |

Figure S5 (continued).

(d) locus 089

| 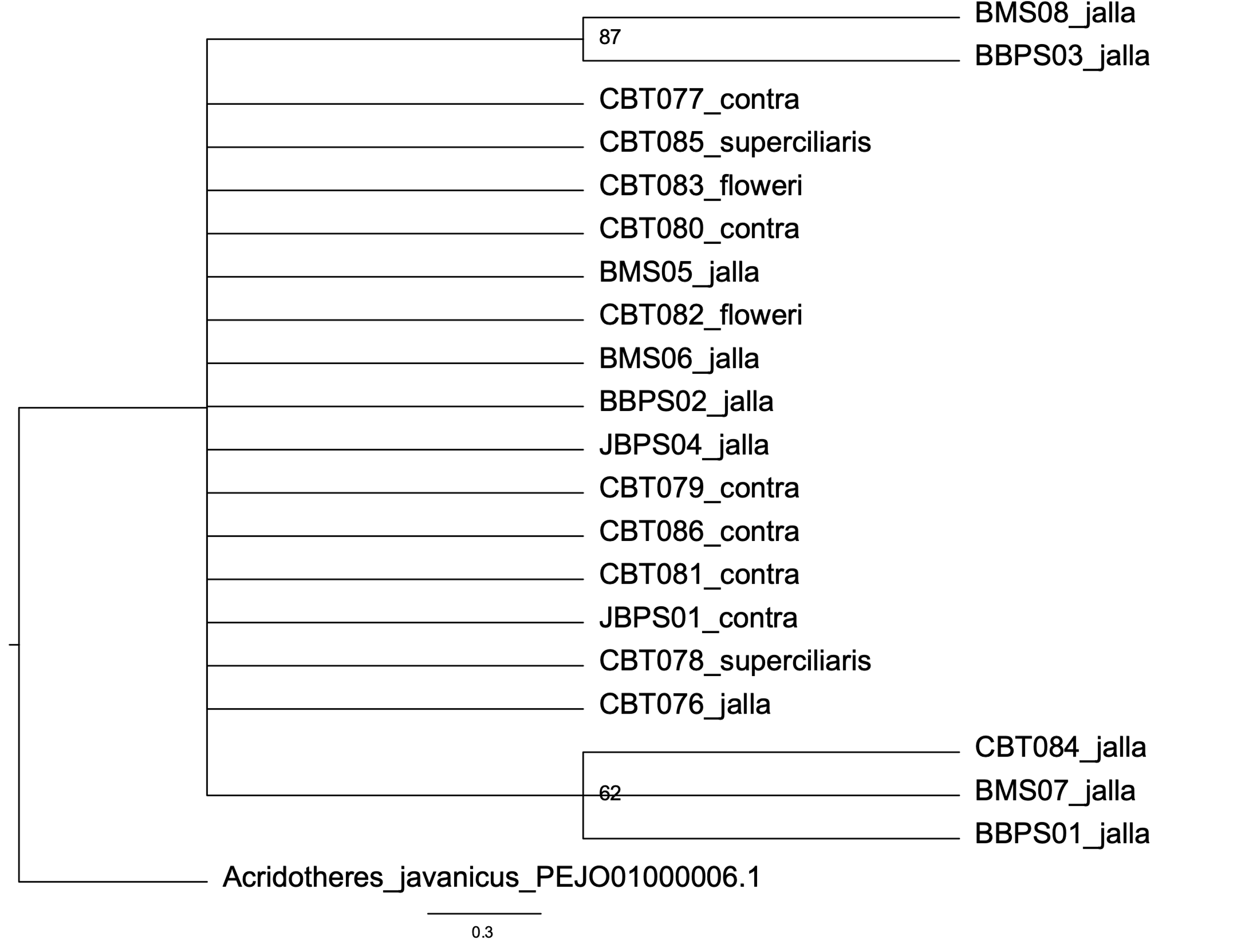 |
| --- |

Figure S5 (continued).

(e) locus 130

| 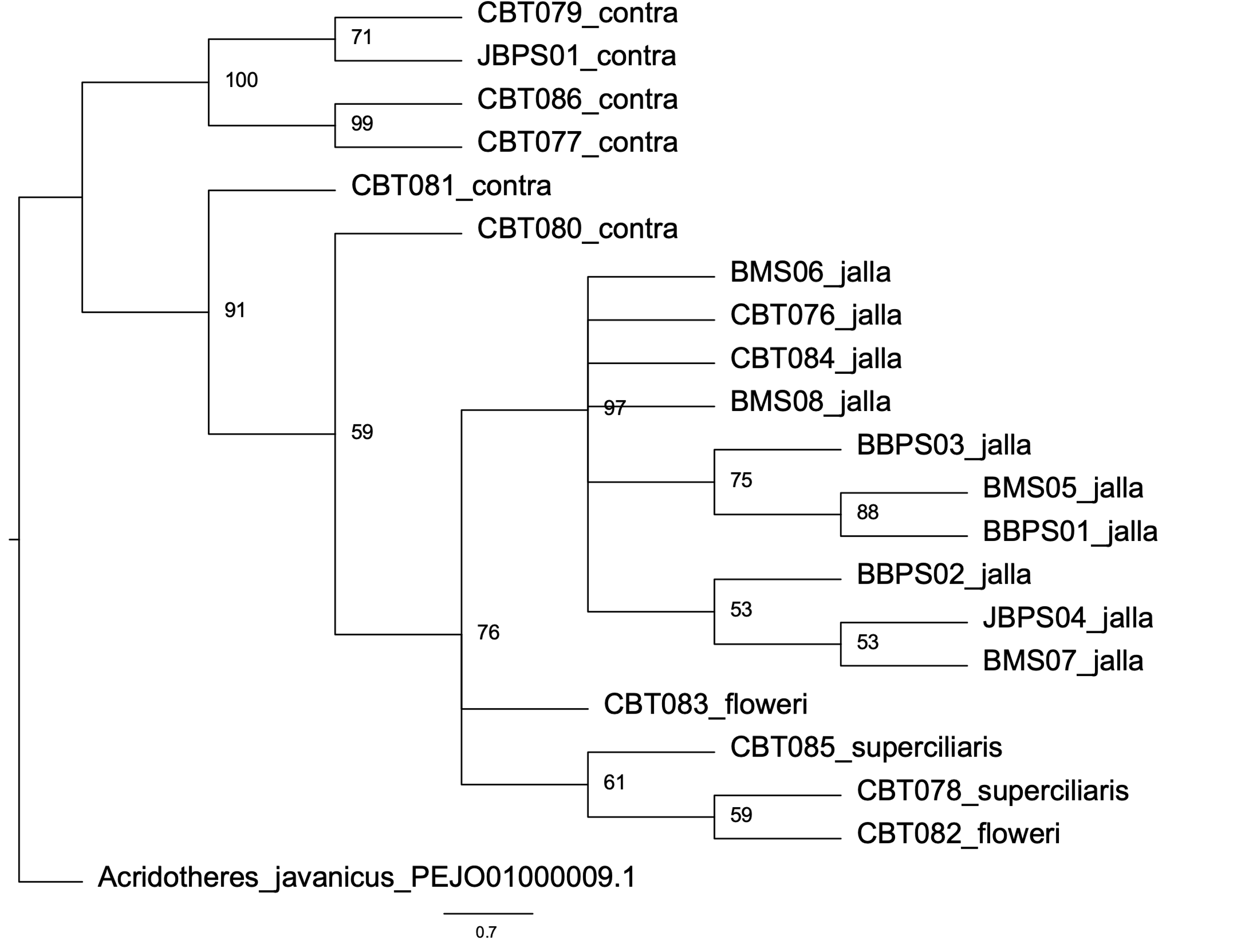 |
| --- |

Figure S5 (continued).

(f) locus 179

| 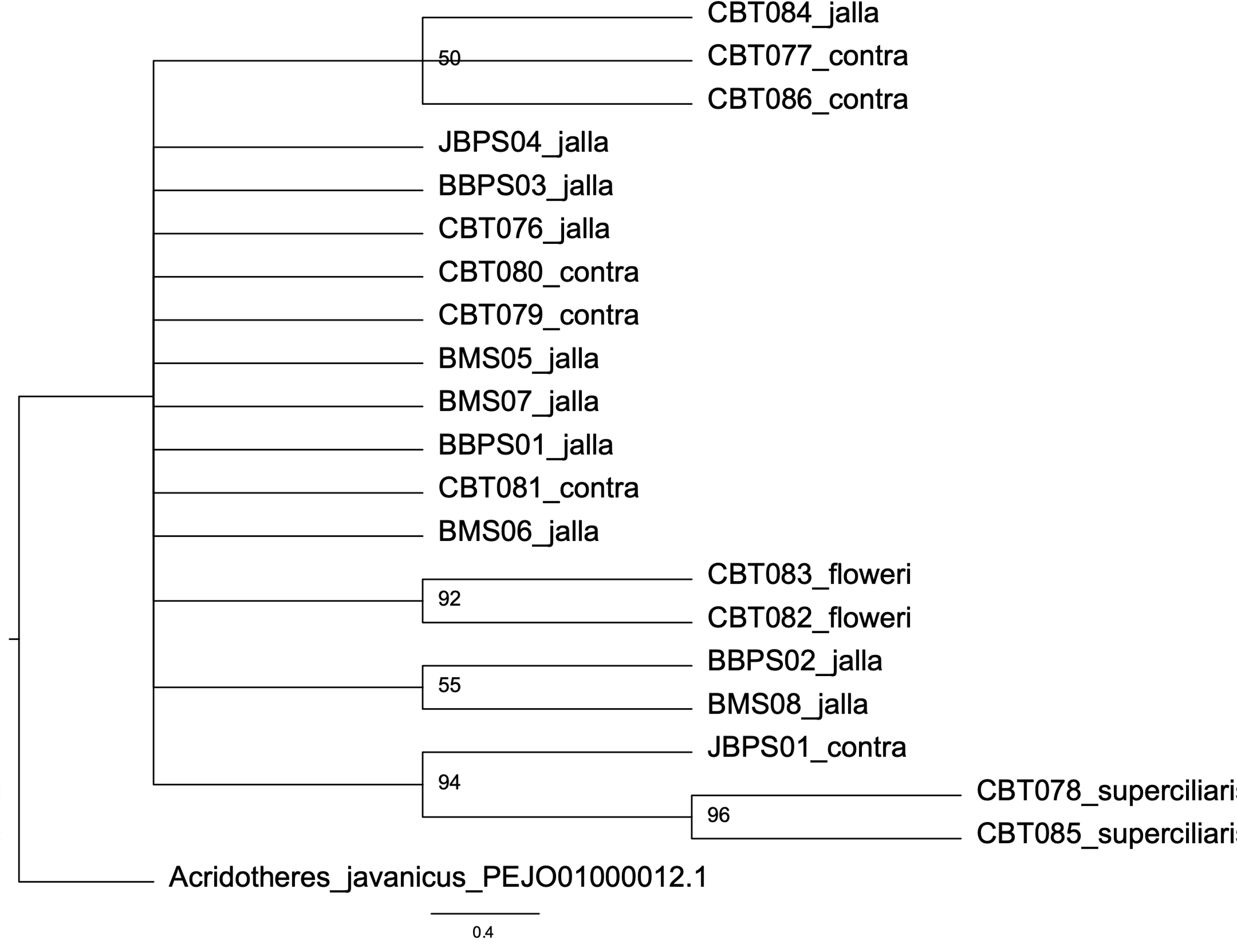 |
| --- |

Figure S5 (continued).

(g) locus 186

| 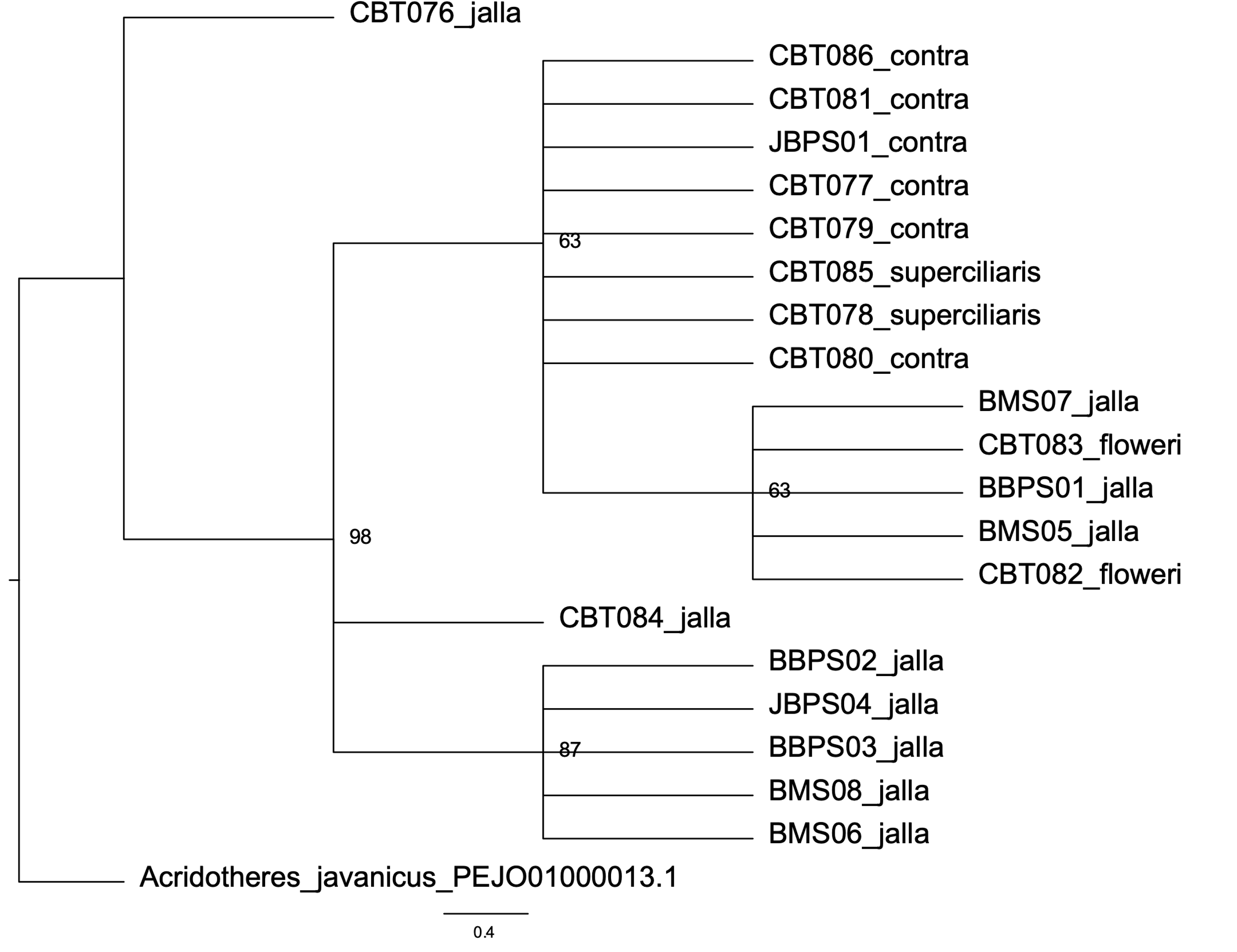 |
| --- |

Figure S5 (continued).

(h) locus 203

193

| 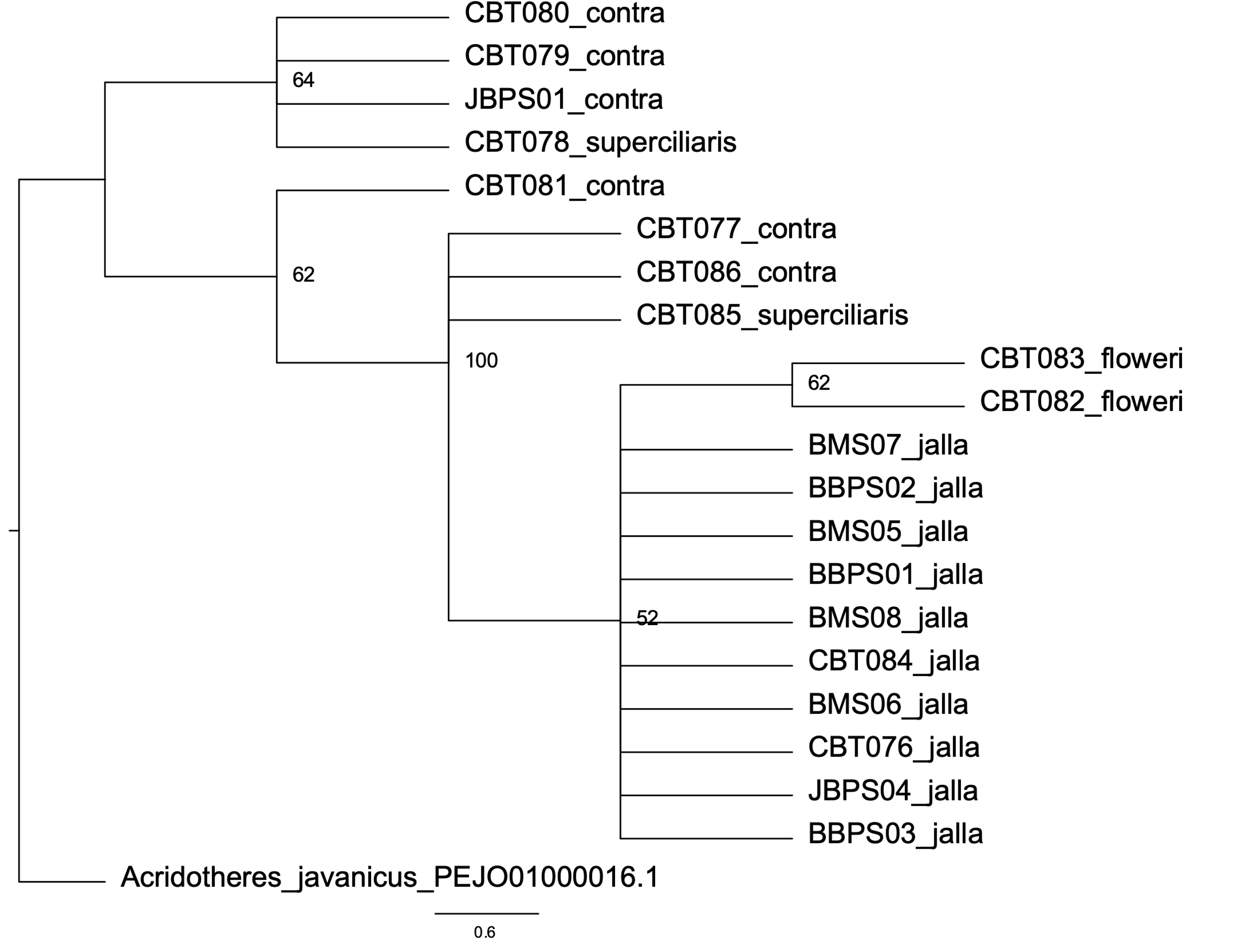 |
| --- |

Figure S5 (continued).

(i) locus 205

| 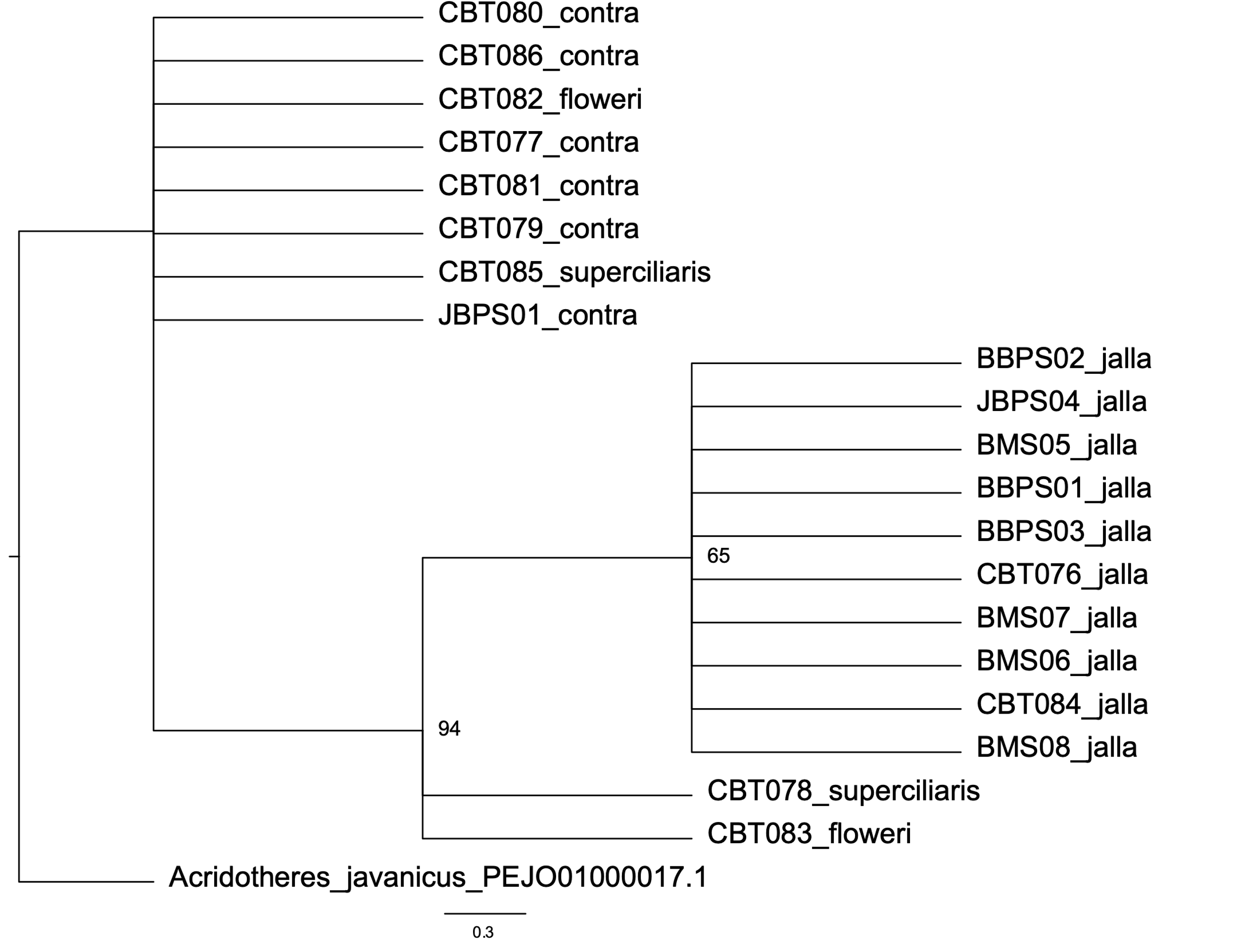 |
| --- |

Figure S5 (continued).

(k) locus 330

| 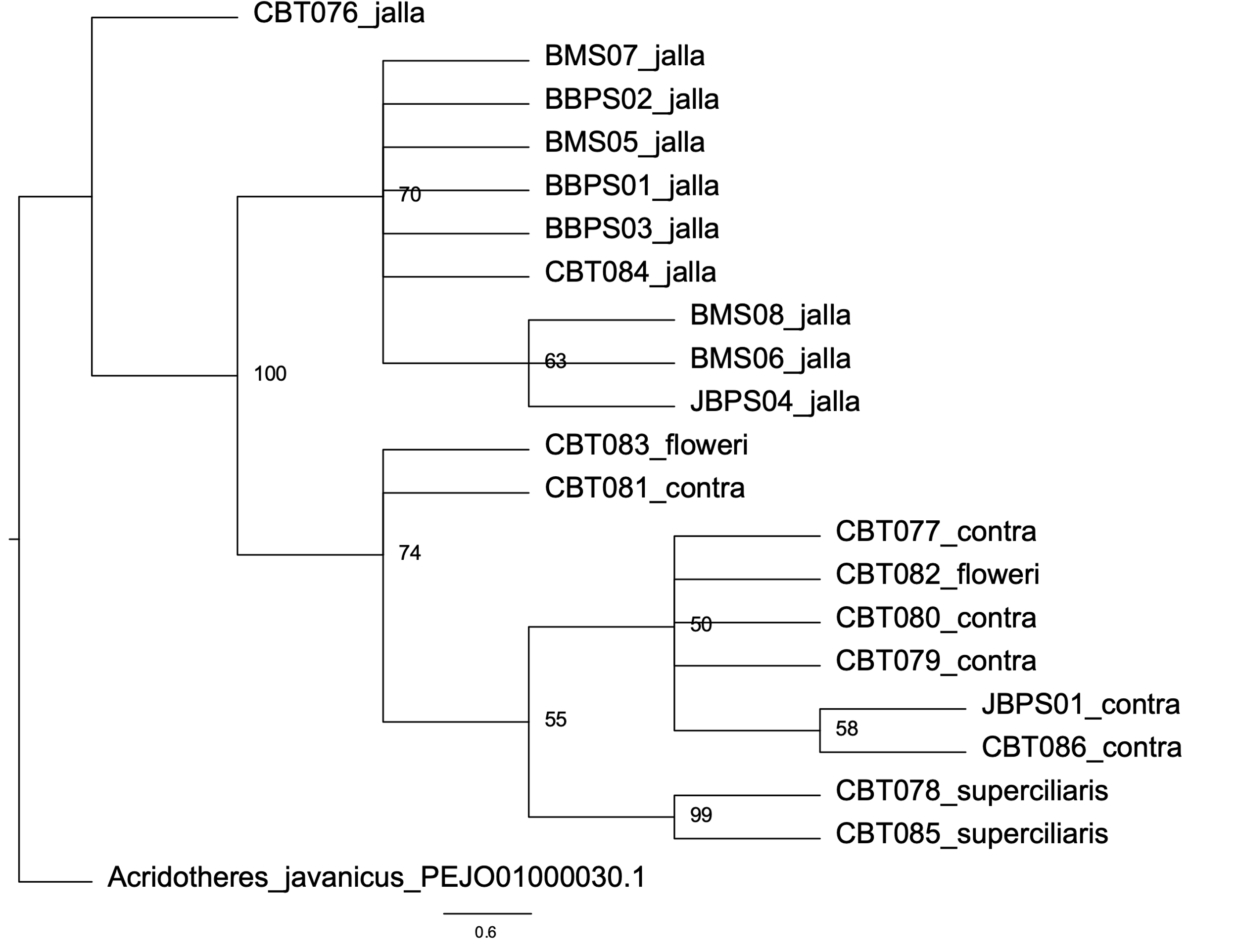 |
| --- |

Figure S5 (continued).

(l) locus 358

| 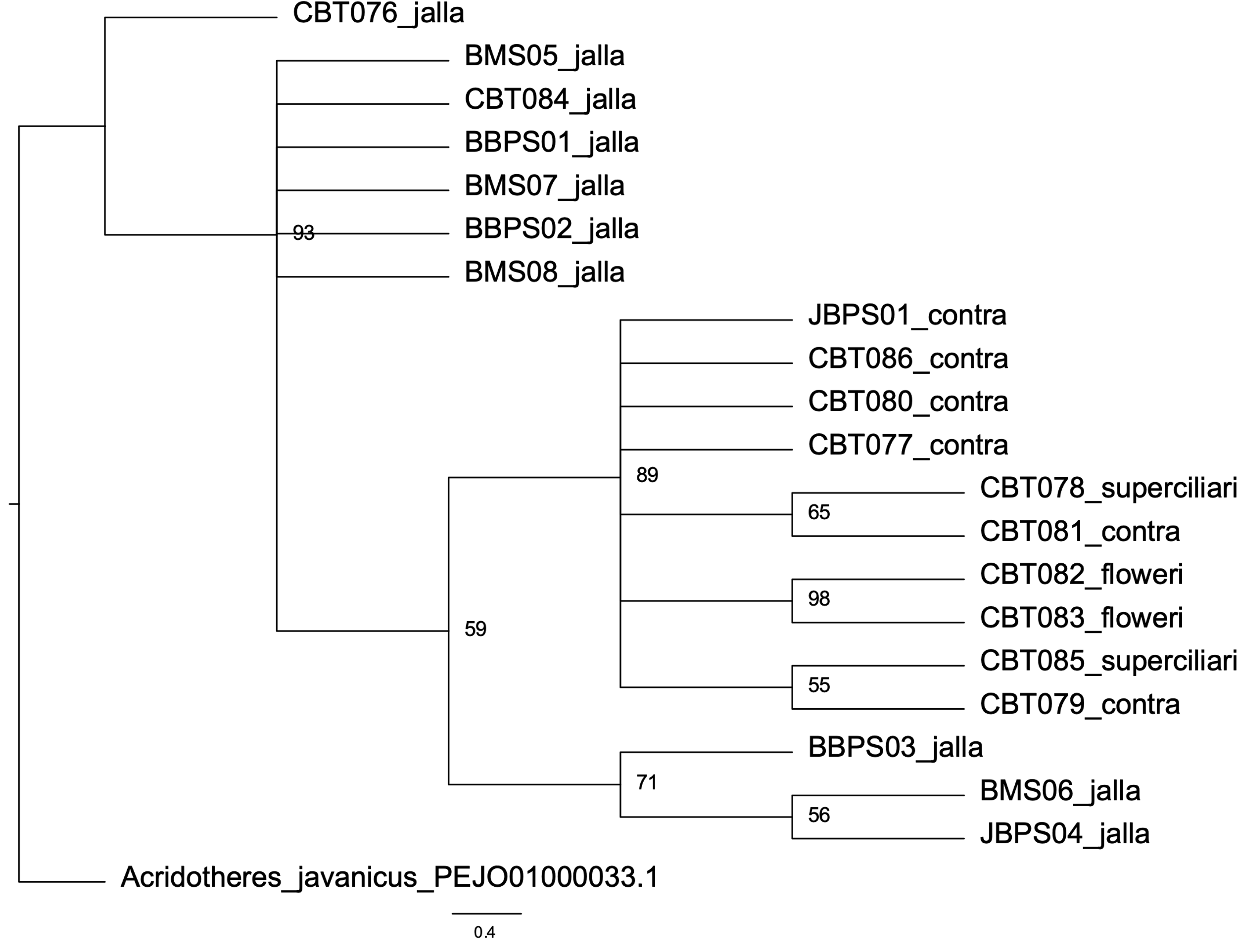 |
| --- |

Figure S5 (continued).

(m) locus 419

| 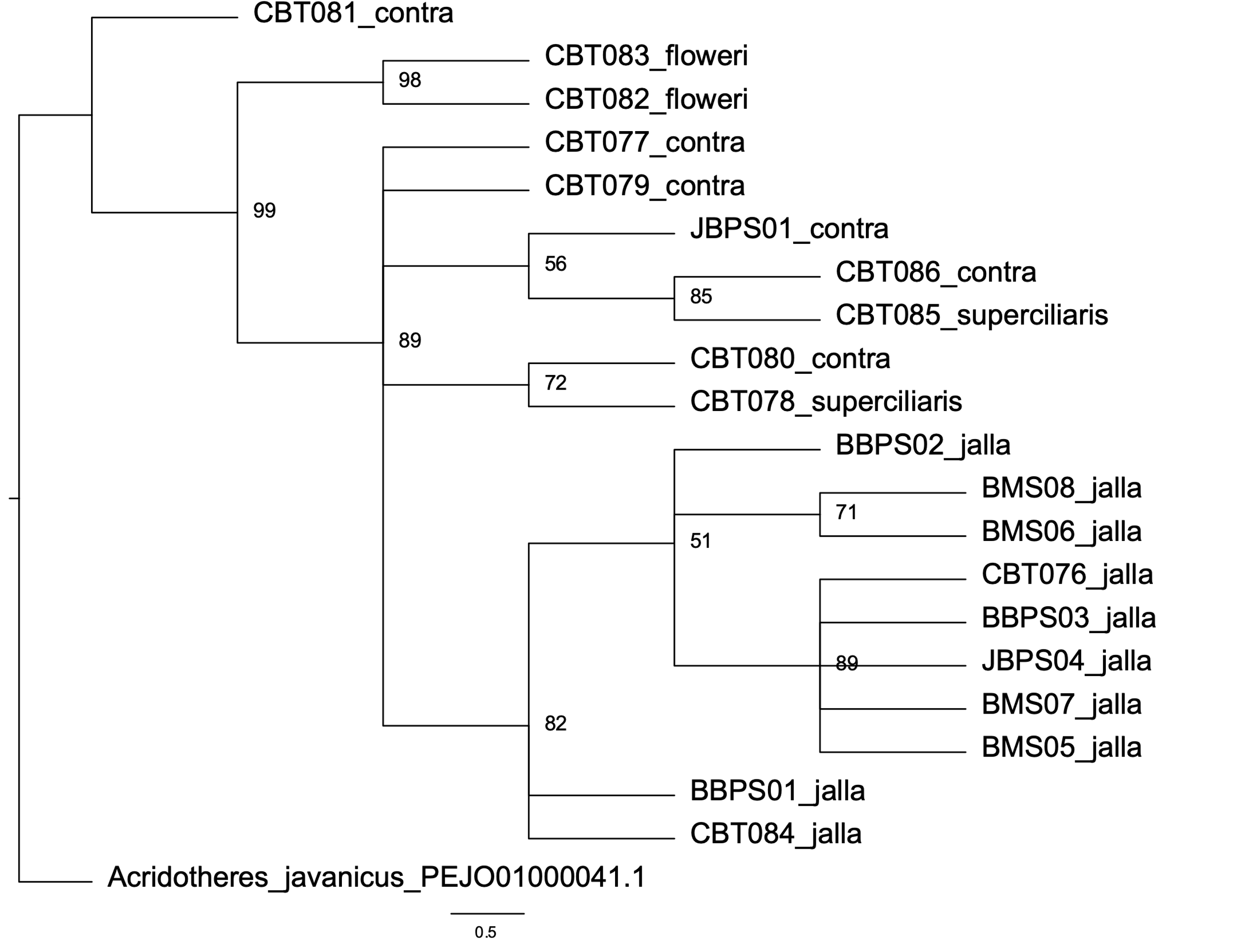 |
| --- |

Figure S5 (continued).

(n) locus 420

| 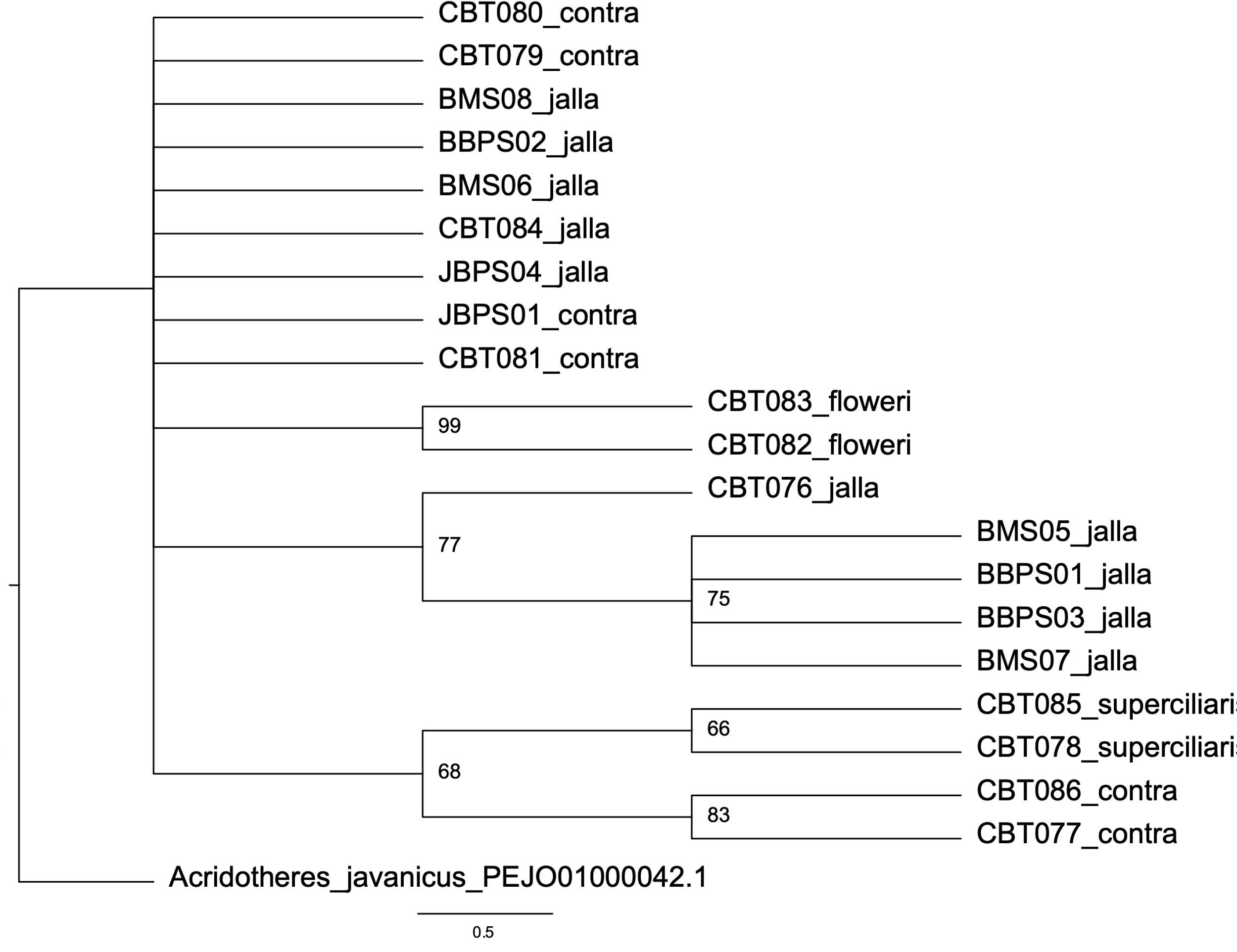 |
| --- |

Figure S5 (continued).

(o) locus 528

| 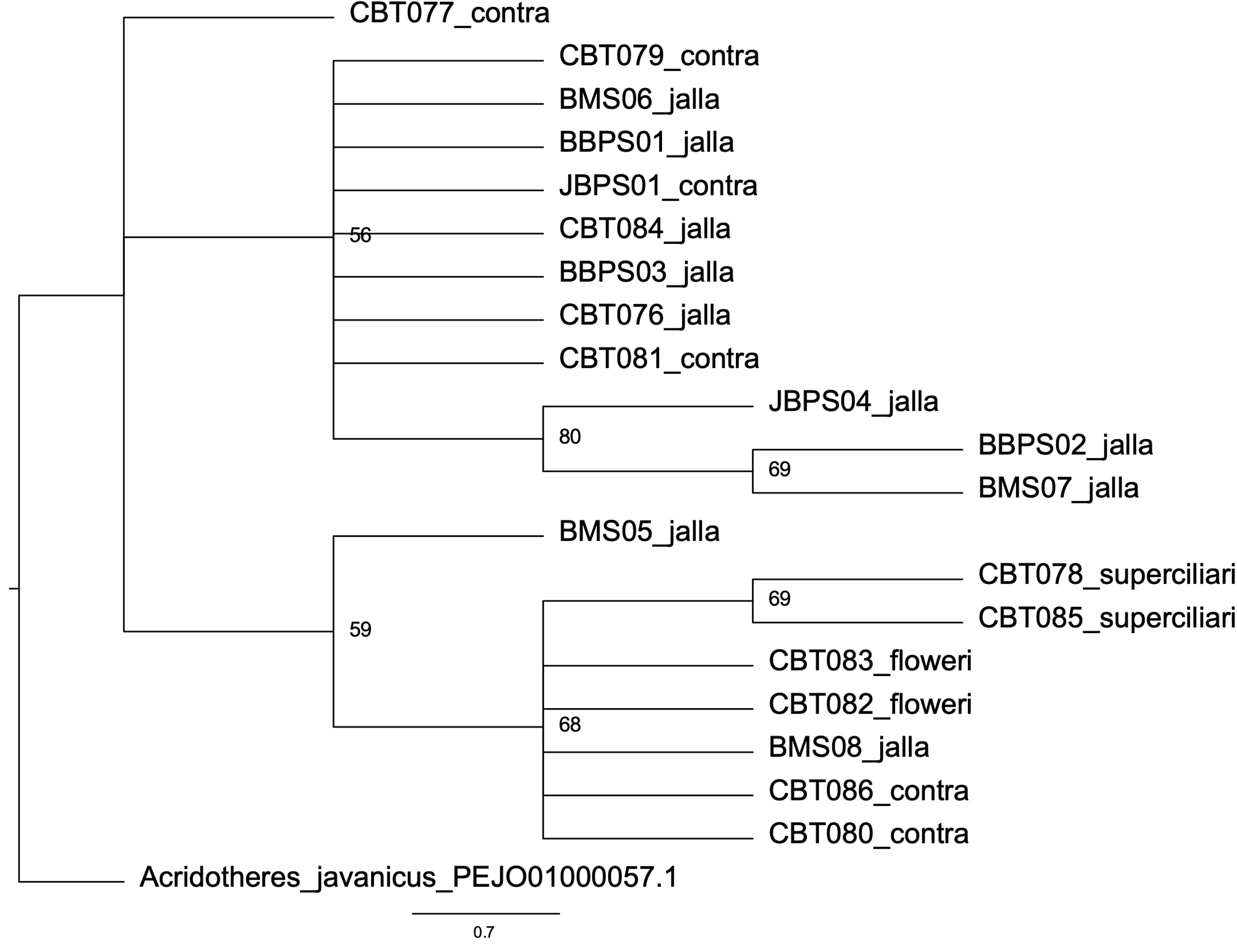 |
| --- |

Figure S5 (continued).

(p) locus 547

| 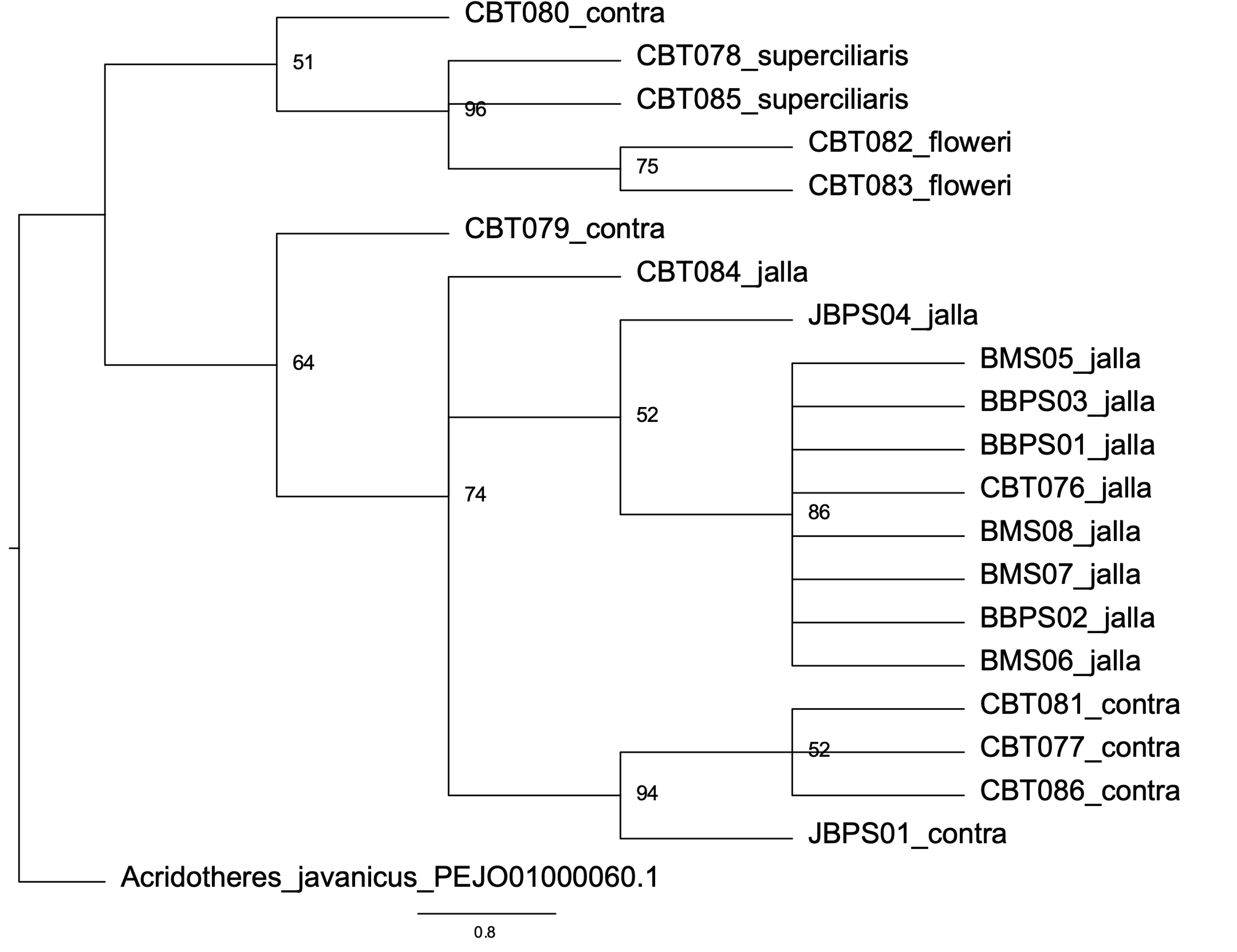 |
| --- |

Figure S5 (continued).

(q) locus 563

| 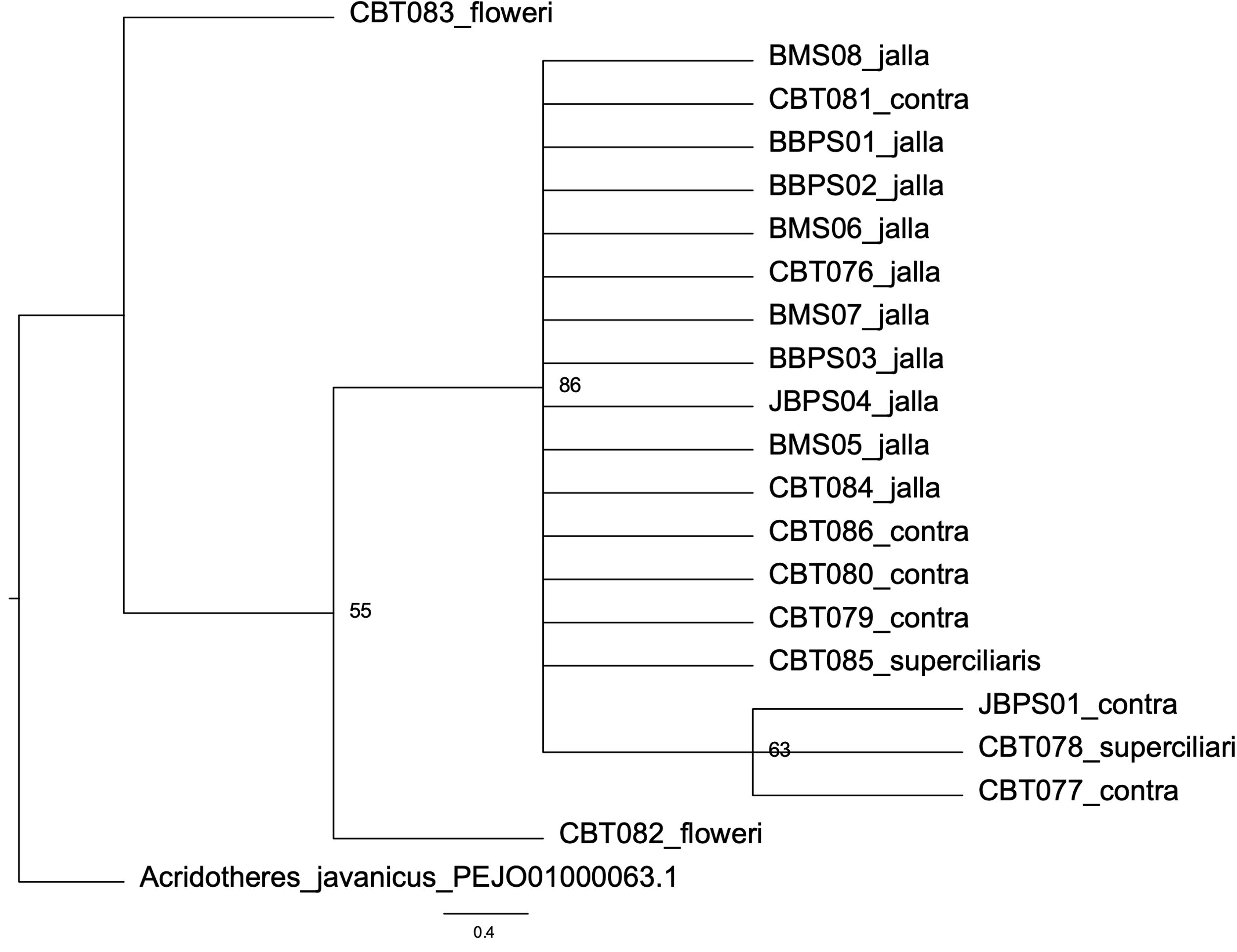 |
| --- |

Figure S5 (continued).

(r) locus 621

| 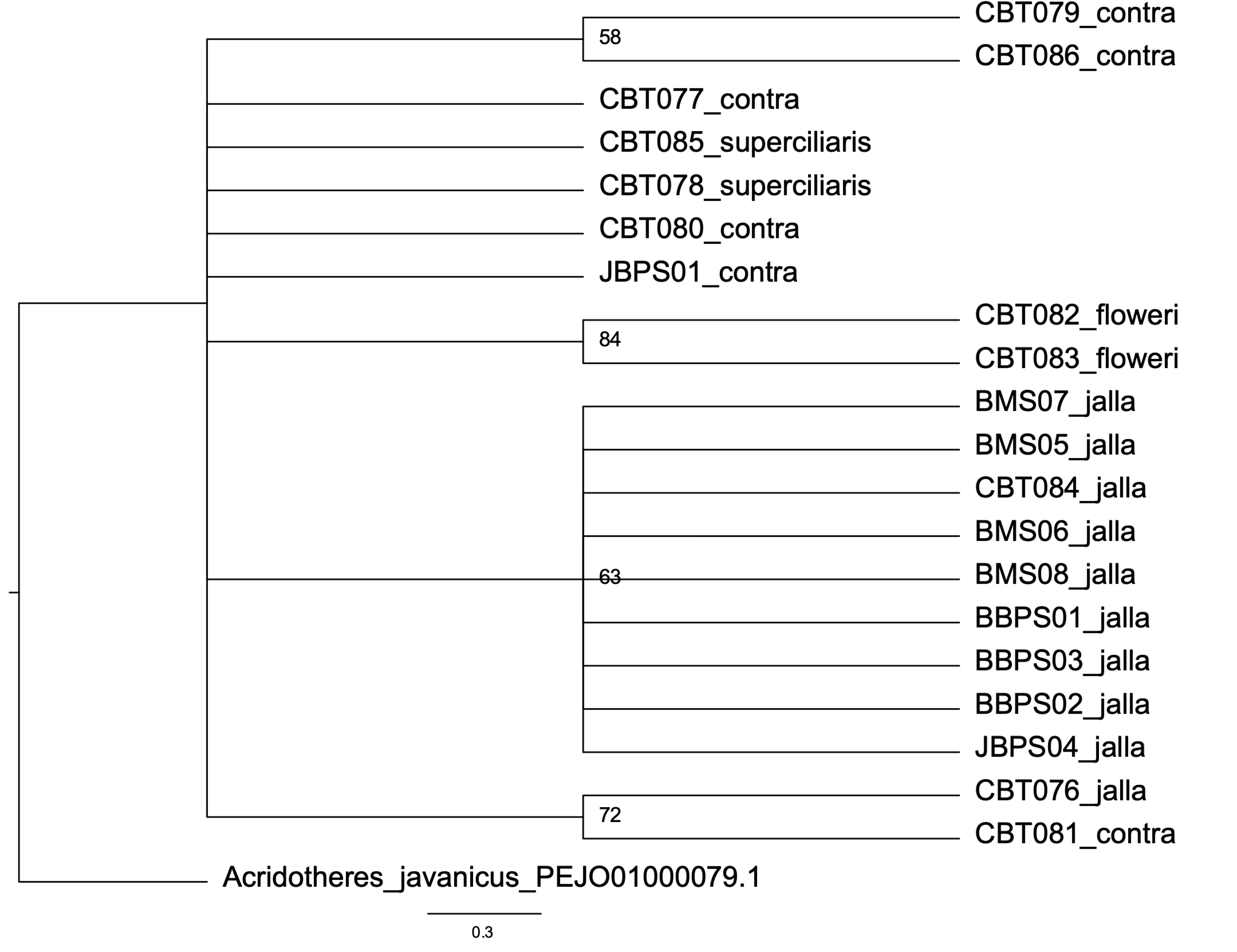 |
| --- |

Figure S5 (continued).

(s) locus 658

| 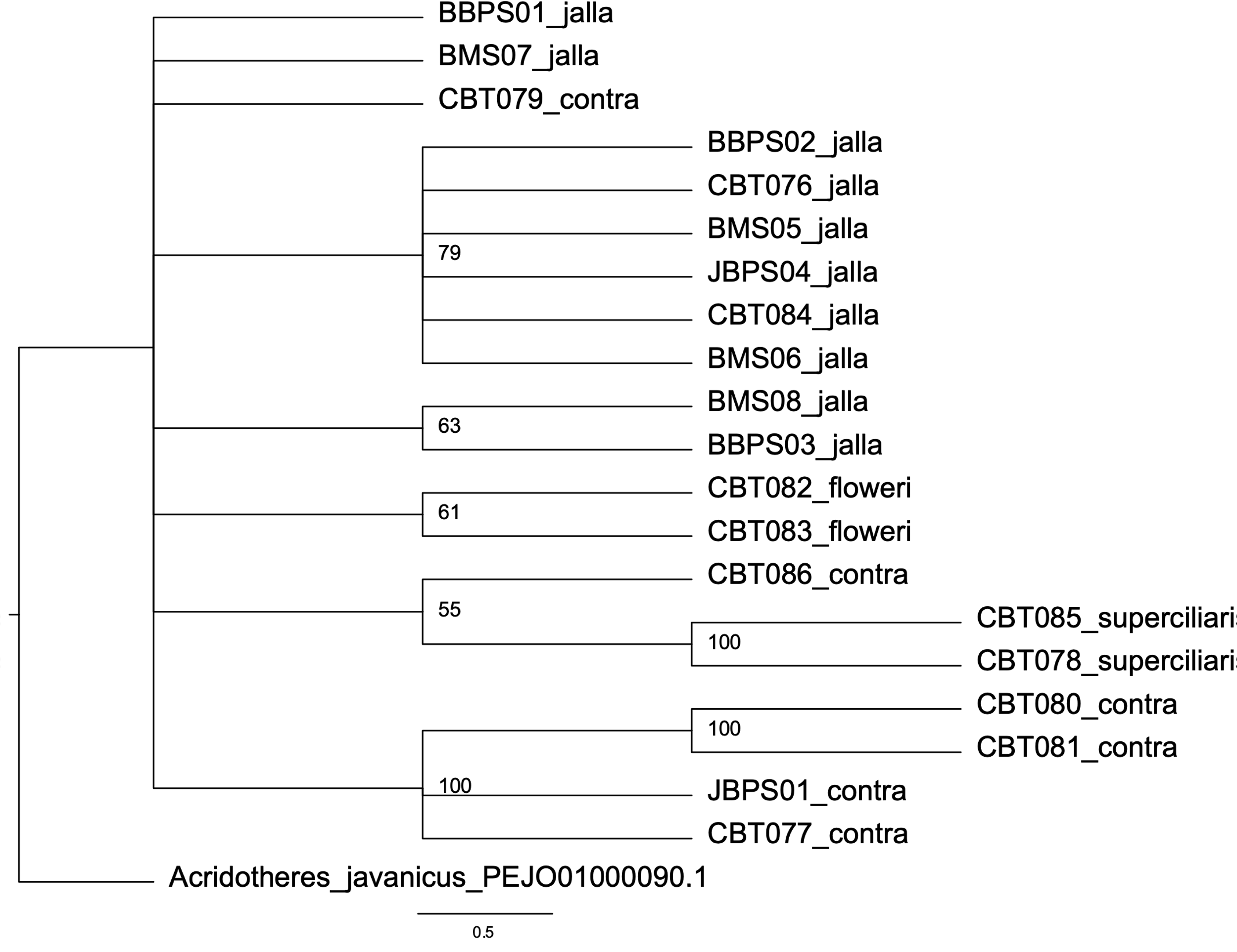 |
| --- |

Figure S5 (continued).

(t) locus 713

| 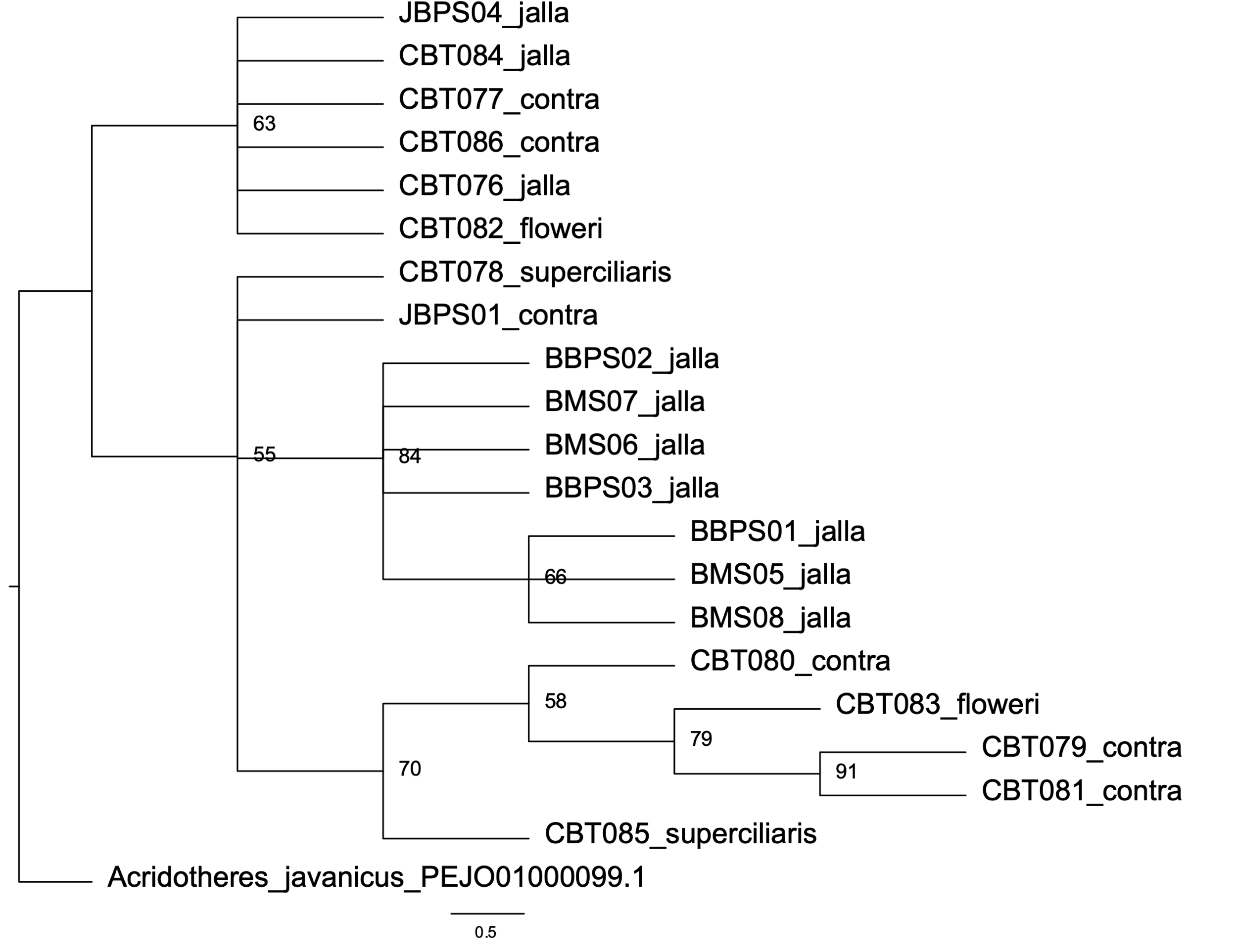 |
| --- |

Figure S5 (continued).

(u) locus 771

| 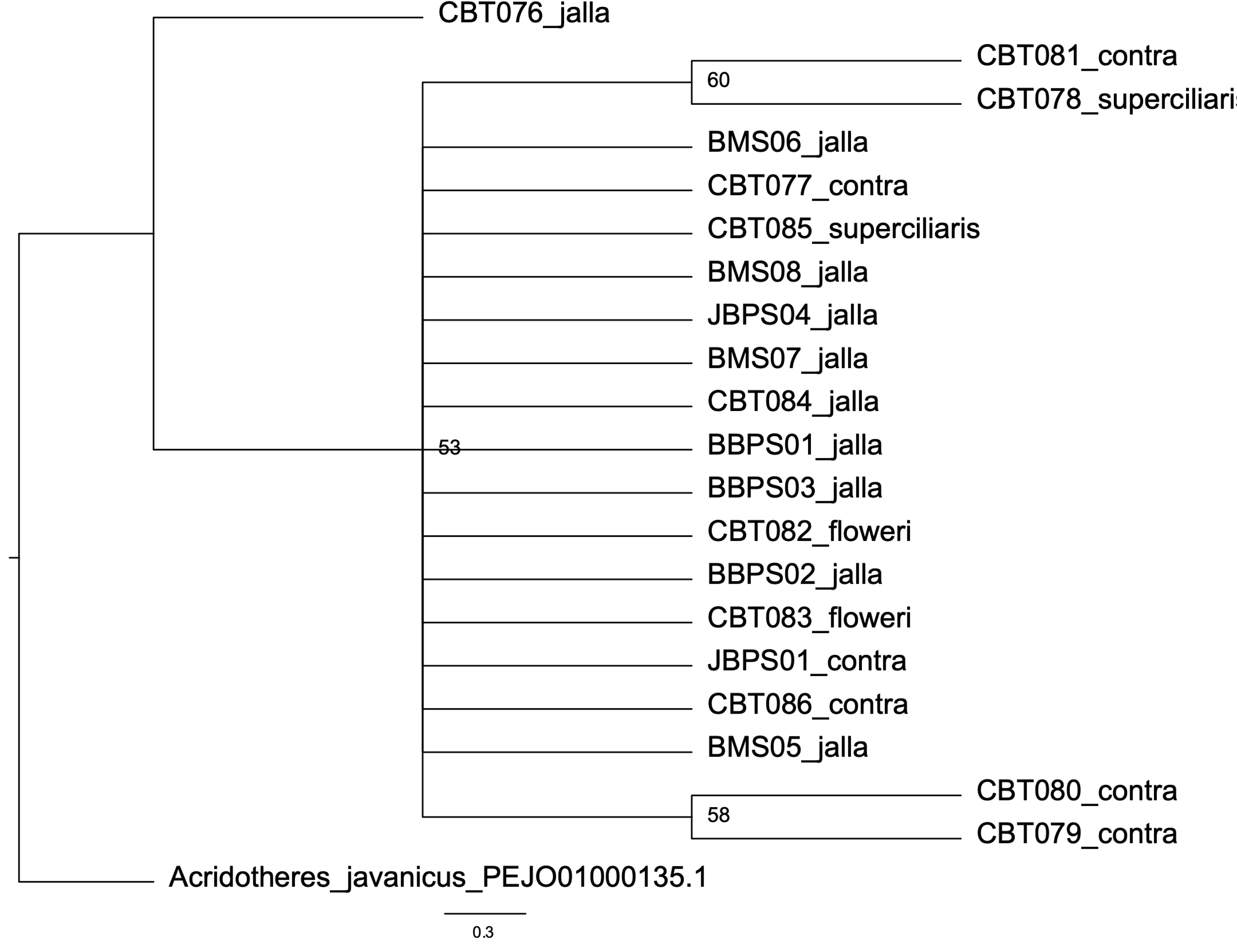 |
| --- |

Figure S5 (continued).

(v) locus 784

| 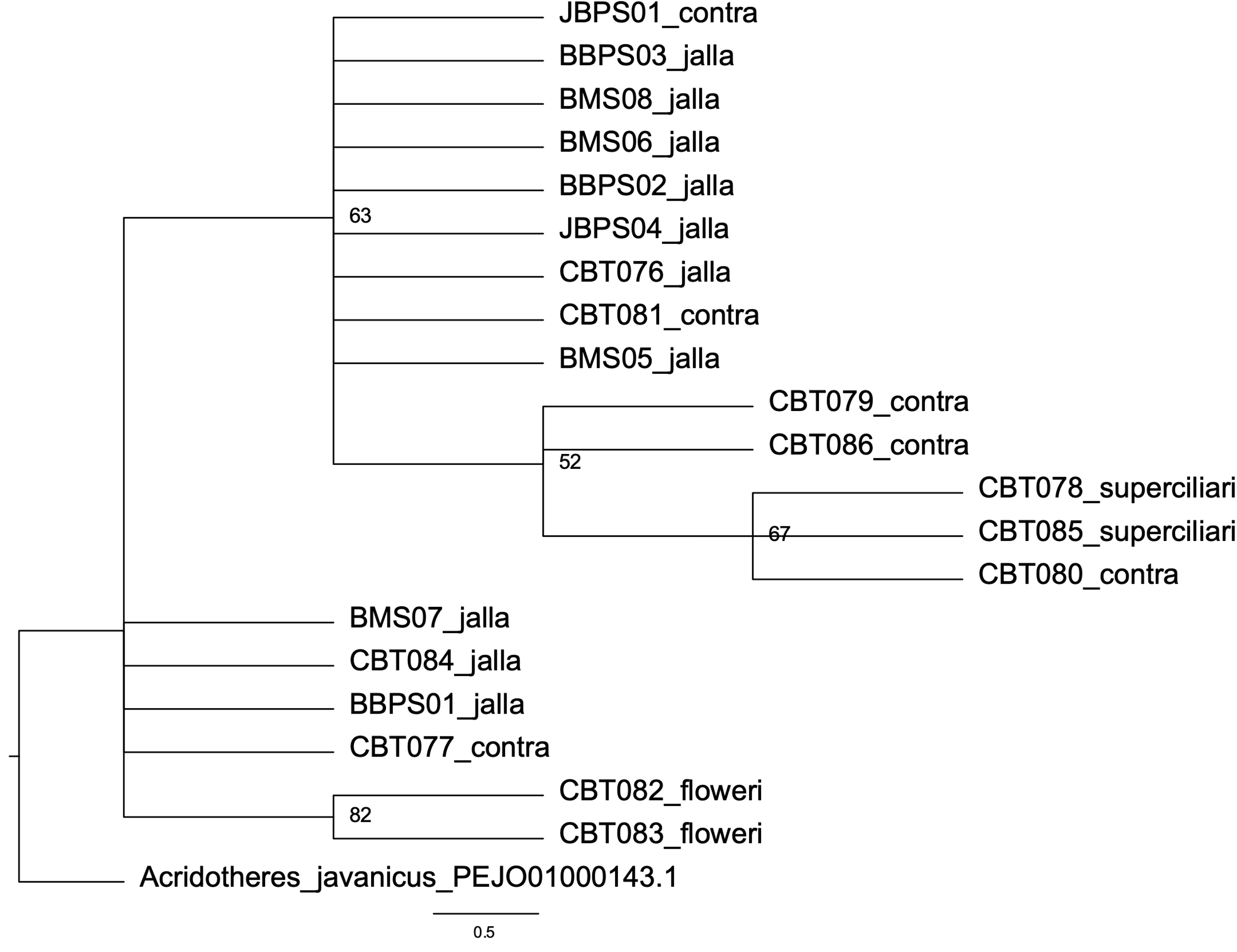 |
| --- |

Figure S5 (continued).

(w) locus 799

| 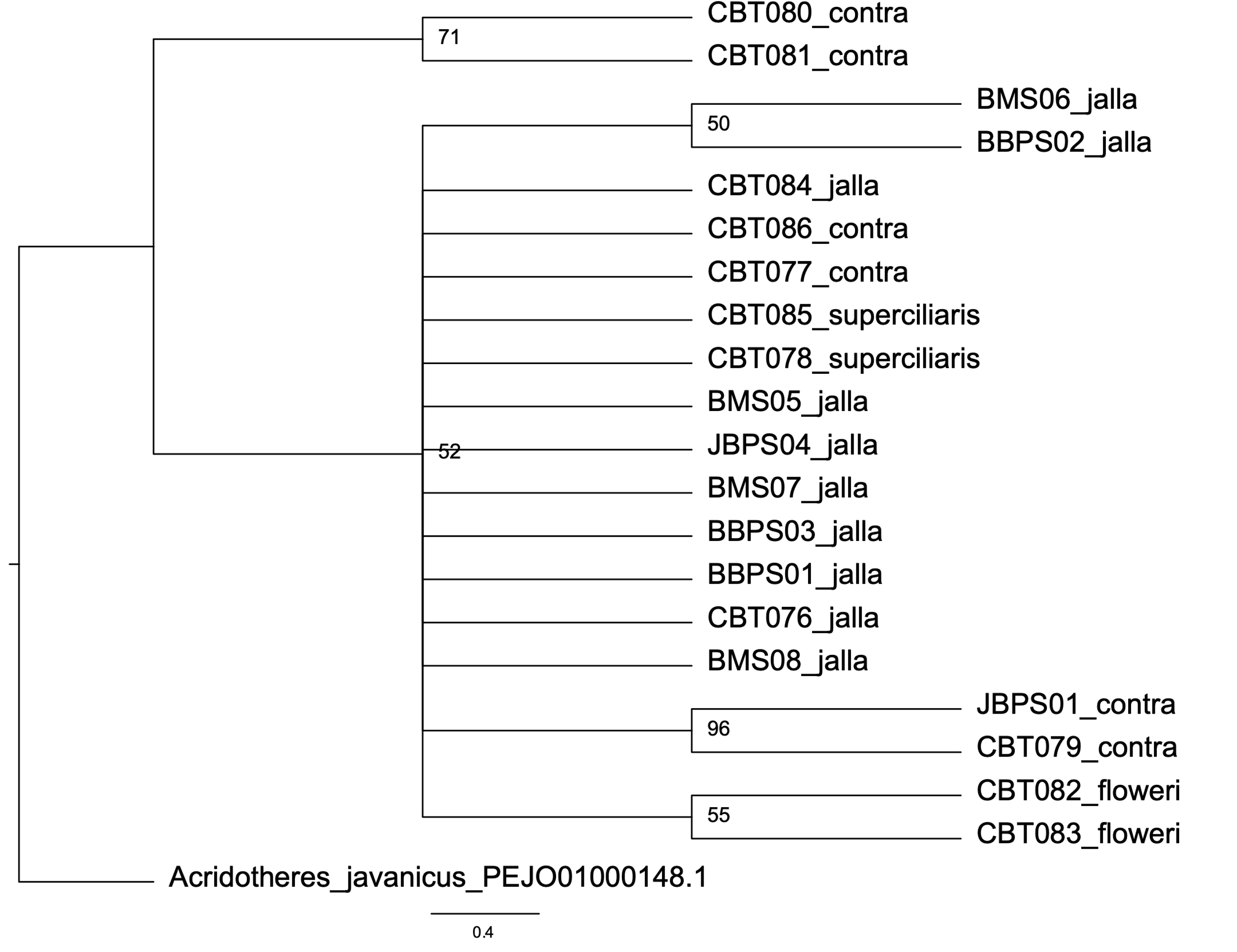 |
| --- |

Figure S5 (continued).

(x) locus 979

| 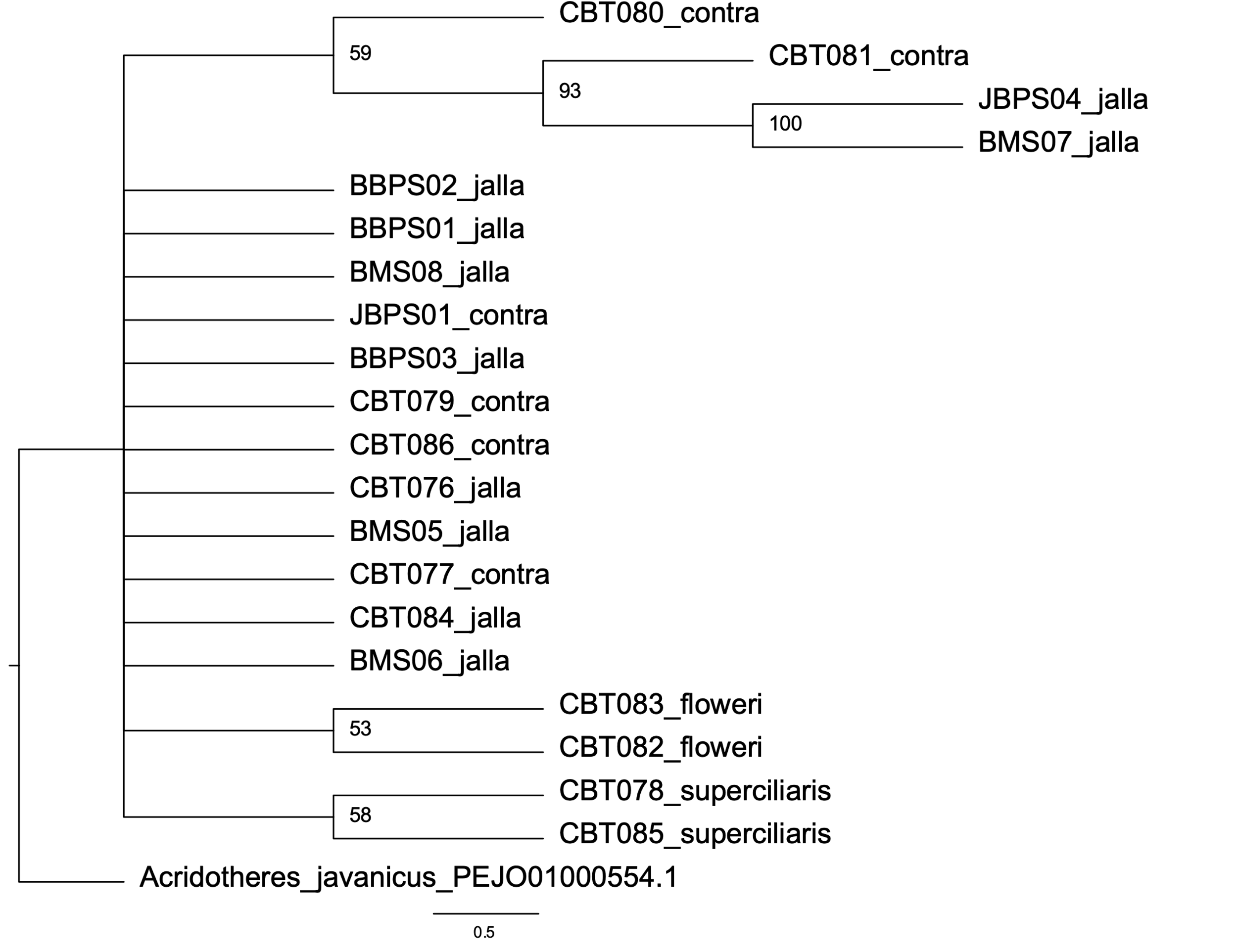 |
| --- |

Figure S5 (continued).

(y) locus 982

| 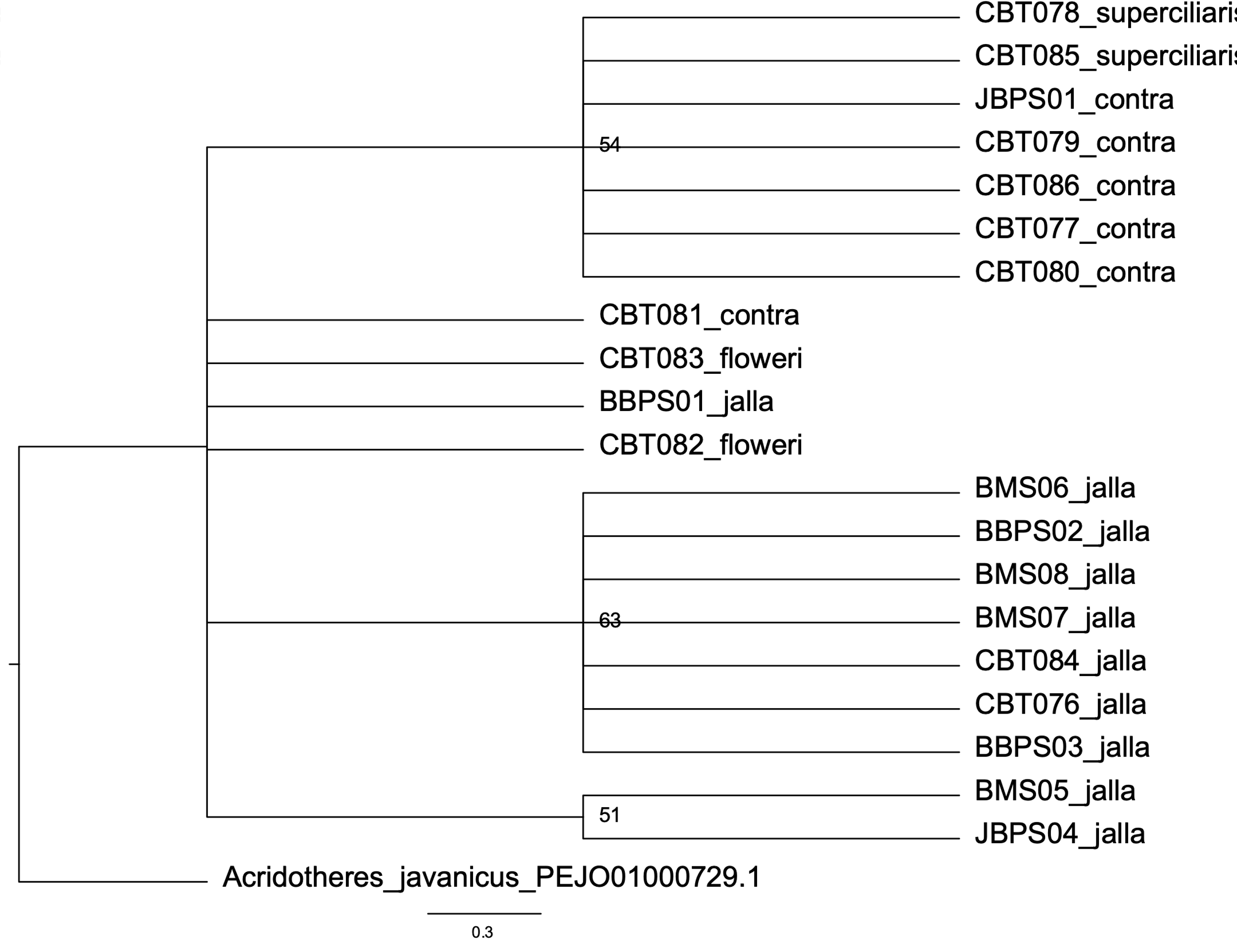 |
| --- |

Figure S5 (continued).

| 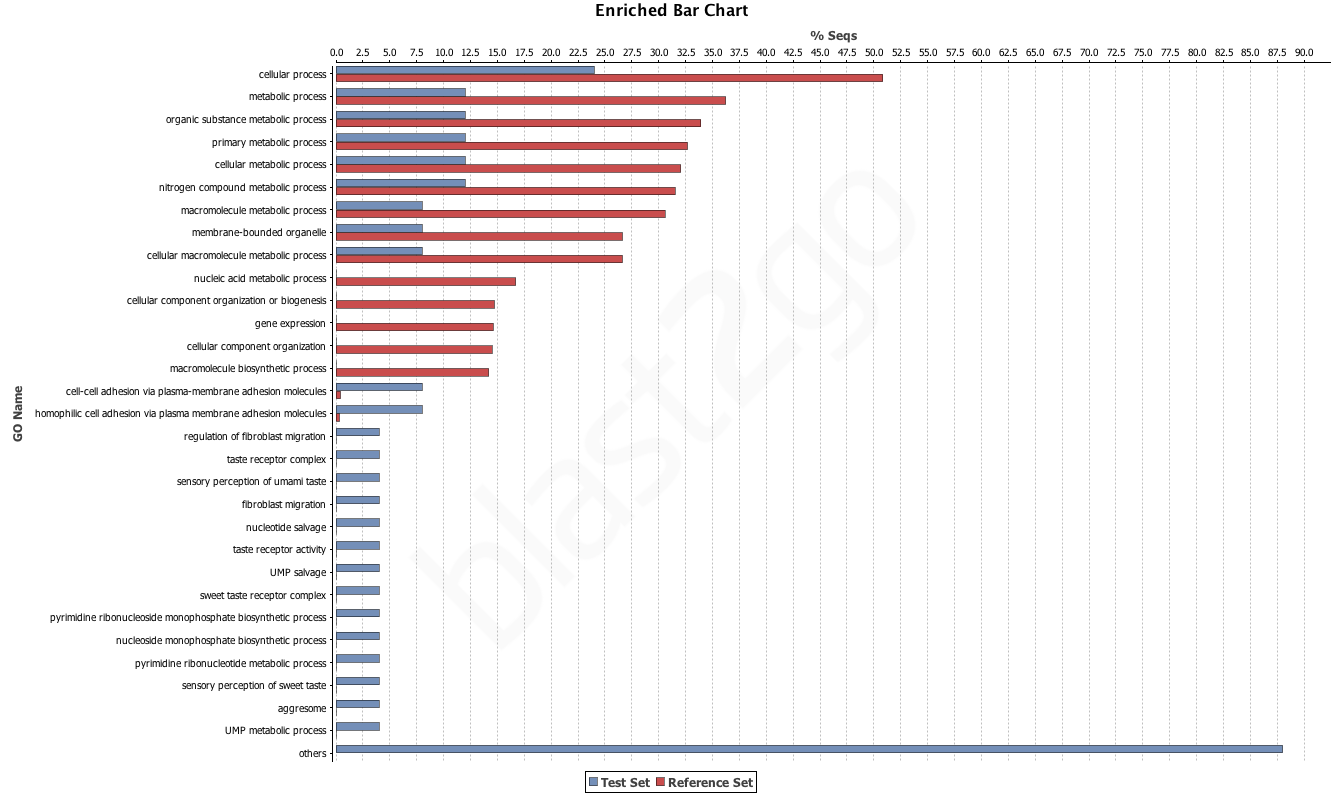 |
| --- |

Figure S6 Bar chart showing the functional categories for the gene ontology (GO) enrichment analysis of a test set of 25 selected candidate loci, each with >20 SNPs per locus, in Blast2Go against the reference set of 980 loci.

The y-axis indicates the GO functional category and the x-axis indicates the relative frequency of each category in the test and reference sets.

| 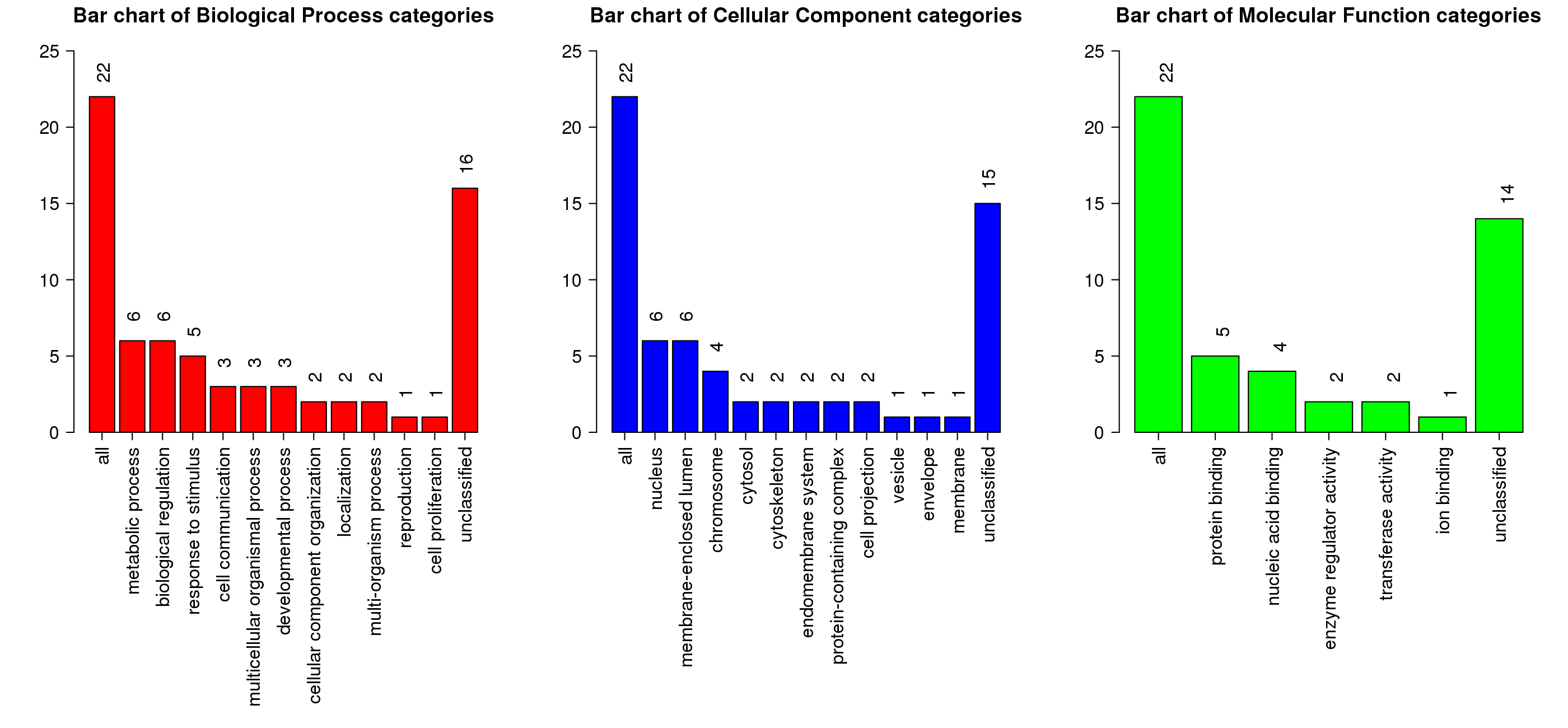 |
| --- |

Figure S7 Bar charts showing the Biological Process, Cellular Component and Molecular Function categories for the test set of 25 selected candidate loci, each with >20 SNPs per locus, against a reference set of 980 loci, using the web-based tool WebGestalt.

The x-axis represents functional categories and the y-axis represents the number of loci in the test set in each of these categories.
